# Identification of a Flavonoid Glucosyltransferase Involved in 7-OH Site Glycosylation in Tea plants (*Camellia sinensis*)

**DOI:** 10.1038/s41598-017-06453-z

**Published:** 2017-07-19

**Authors:** Xinlong Dai, Juhua Zhuang, Yingling Wu, Peiqiang Wang, Guifu Zhao, Yajun Liu, Xiaolan Jiang, Liping Gao, Tao Xia

**Affiliations:** 1 State Key Laboratory of Tea Plant Biology and Utilization, Anhui Agricultural University, Hefei, Anhui 230036 China; 2 School of Life Science, Anhui Agricultural University, Hefei, Anhui 230036 China

## Abstract

Flavonol glycosides, which are often converted from aglycones in a process catalyzed by UDP-glycosyltransferases (UGTs), play an important role for the health of plants and animals. In the present study, a gene encoding a flavonoid 7-*O*-glycosyltransferase (CsUGT75L12) was identified in tea plants. Recombinant CsUGT75L12 protein displayed glycosyltransferase activity on the 7-OH position of multiple phenolic compounds. In relative comparison to wild-type seeds, the levels of flavonol-glucosides increased in Arabidopsis seeds overexpressing *CsUGT75L12*. In order to determine the key amino acid residues responsible for the catalytic activity of the protein, a series of site-directed mutagenesis and enzymatic assays were performed based on the 3D structural modeling and docking analyses. These results suggested that residue Q54 is a double binding site that functions as both a sugar receptor and donor. Residues H56 and T151, corresponding to the basic active residues H20 and D119 of VvGT1, were not irreplaceable for CsUGT75L12. In addition, residues Y182, S223, P238, T239, and F240 were demonstrated to be responsible for a ‘reversed’ sugar receptor binding model. The results of single and triple substitutions confirmed that the function of residues P238, T239, and F240 may substitute or compensate with each other for the flavonoid 7-*O*-glycosyltransferase activity.

## Introduction

UDP-glycose:flavonoid glycosyltransferases (UFGTs), which are encoded by a multigene family, catalyze the biosynthesis of glycosylated phenolic compounds in plants. The glycosylation of phenolic compounds imparts specific biochemical and physiological properties to these compounds, which allows them to participate in different types of biochemical reactions. For example, the glycosylated phenolic compounds enable plants to better cope with biotic and abiotic stress^1–4^.

Since only a few of the genes responsible for the biosynthesis of glycosylated phenolic compounds have been identified in tea plants, our understanding is limited with respect to synthesized compounds and their respective physiological roles in tea plants. In our previous report, 132 putative CsUGTs proteins were identified among the various transcriptome datasets deposited in the NCBI database (https://blast.ncbi.nlm.nih.gov/Blast.cgi). Subsequently, sequence analyses revealed a clustering into 16 phylogenetic groups (A to M, O, P, and R)^5^. Among the identified proteins, CsUGT84A22 exhibited catalytic activity toward phenolic acids, including gallic acid. β-glucogallin (βG), a glucose ester of gallic acid, acts as an efficient acyl donor during the biosynthesis of galloylated catechins^6^. Both CsUGT78A14 and CsUGT78A15 were identified as glycosyltransferases that catalyze the glycosylation of flavonoids at the 3-OH position using UDP-glucose and UDP-galactose as sugar donors^5^. Flavonol 3-*O*-glucoside and flavonol 3-*O*-galactoside derivatives are known as the principal components responsible for the velvety, astringent flavor of tea beverages^7, 8^.

In order to better understand the biosynthesis and transport of polyphenols in tea plants, additional studies of UFGTs are warranted. For example, the regulation of the epicatechin 3′-*O*-glucoside and cyanidin 3-*O*-glucoside^9^, which serve as substrates for MATE or ABC transporters^10^, may involve the transportation of phenolic compounds from the ER to the vacuole^9^. The genes responsible for the biosynthesis of flavonoid 7-*O*-glycoside and the involvement of these compounds in plants responsing to stress and increased resistance to pathogens, have been identified in some plants^11^. To date, the specific function of homologous genes in tea plants has not been elucidated. The large number and structural diversity of the UDP-glycosyltransferase multigene family makes this task difficult and complex.

UDP-glycosyltransferases utilize highly divergent sugar donors, as well as sugar acceptors^12^. The most common sugar donors; recognized by plant UDP-glycosyltransferases include UDP-glucose, UDP-galactose, UDP-glucuronic acid, UDP-xylose, UDP-mannose, and UDP-rhamnose. The major glucosylation sites residue at the 3-, 5-, 7-, 3′-, 4′-OH positions of flavonoid sugar acceptors^5, 13–21^. Among 91 recombinant UGTs analyzed in *Arabidopsis thaliana*, 29 exhibited significant catalytic activity toward quercetin; of which eleven catalyzed the glucosylation at the 3-OH position. Only three utilized the 7-OH group position and the other fifteen catalyzed glucosylation at multiple positions^3, 14^. Over the last several decades, the acceptor recognition and sugar-donor selectivity of UDP-glycosyltransferases in plants has been the subject of extensive investigation^12, 22^.

While the sequences of a large number of plant UGT genes are available in public databases, only a few crystal structures of UGT proteins have been reported^13, 22–25^. The gap between the abundant available resource of existing sequences and a limited number of crystal structures has driven the interest in developing computational methods for predicting protein structures^26^. Homology modeling and protein-ligand molecular interaction docking analyses, based on the crystal structure databases, along with site-directed rational mutagenesis studies have become powerful tools for predicting functional residues in proteins^26–28^. For example, 3D structure modeling and virtual docking with ligands is currently available for the rapid prediction of functional residues. The key amino acids involved in the catalytic mechanism of flavonoid 3-*O*-glycosyltransferase were illustrated by solved crystal structures of the plant UGTs, UGT78G1 and UGT85H2 from M. truncatula and VvGT1 from V. v inifera^13, 24, 25^. Little is known, however, regarding to the amino acids that play key functional roles in flavonoid 7-*O*-glycosyltransferase activity.

In the present study, *CsUGT75L12* was identified in tea plants (*Camellia sinensis*) and confirmed to a flavonoid 7-*O*-glycosyltransferase. A reverse genetic approach was used to investigate the properties of *CsUGT75L12 in vitro* and *in planta*. Homology modeling and a series of site-directed mutagenesis manipulations were performed in order to determine the key amino acids contributing to flavonoid 7-*O*-glucosyltransferase activity.

## Results

### Isolation of CsUGT75L12 from tea plants

A full-length *CsUGT75L12* cDNA was synthesized based on sequence obtained from the results of the 3′- and 5′-RACE PCR using an *EST* sequence for primer design. Results indicated that the size of the 3′ and 5′ non-coding regions and the full-length of cDNA were 161 bp, 269 bp and 1966bp, respectively(Suppl Figure S1). The open reading frame (1536 bp) of the cDNA encodes a putative 52,035 kDa protein. The cDNA sequence was submitted to NCBI (accession KP682364) and the gene, *CsUGT75L12*, was named according to the naming conventions set by the UGT nomenclature committee (http://www.flinders.edu.au/medicine/sites/clinical-pharmacology/ugt-homepage.cfm).

**Figure 1:**
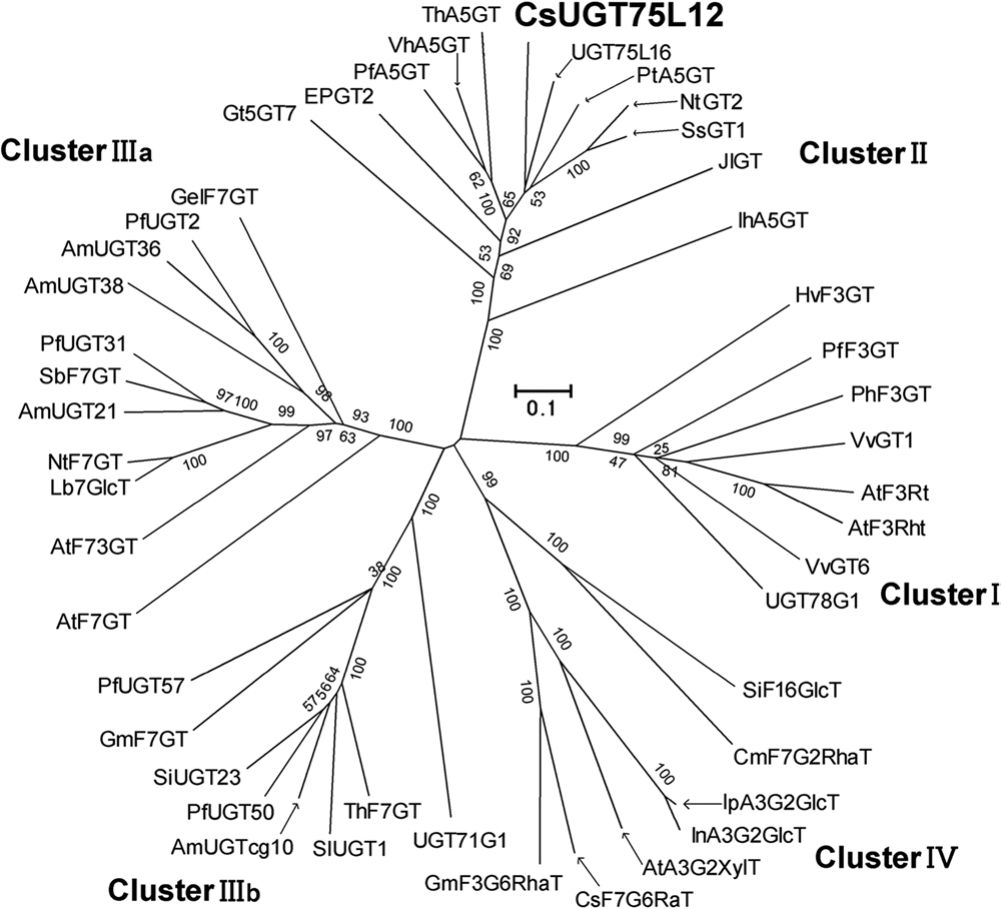
Phylogenetic tree of CsUGT75L12 and flavonoid UGTs. An unrooted phylogenetic tree was constructed using Mega 5.0 software with the neighbor-joining method. CsUGT75L12 was identified in this study and functionally characterized as flavonoid UGTs. The Phylogenetic tree was classified into five clusters (I, II, IIIa, IIIb and IV) based on the different enzymatic function.

With the use of MEGA5.0 software, a phylogenetic tree was constructed, using CsUGT75L12 along with previously identified flavonoid UGTs with known function, resulting in the formation of five clusters (cluster I, cluster II, clusterIIIa, clusterIIIb, and cluster IV) (Fig. 1). CsUGT75L12 was placed into cluster II with flavonoid 5-*O*-glycosyltransferases, such as VhA5GT^29^; anthocyanin 5-*O*-glycosyltransferases, such as PfA5GT and IhA5GT^30, 31^; flavonoid 7-*O*-glycosyltransferases, such as SsGT1 and NTGT2^11, 32^; and a specific protein, UGT75L6, from *Gardenia jasminoides*, which preferentially glucosylates the carboxyl group of crocetin to yield crocetin glucosyl esters^33^.

**Figure 2:**
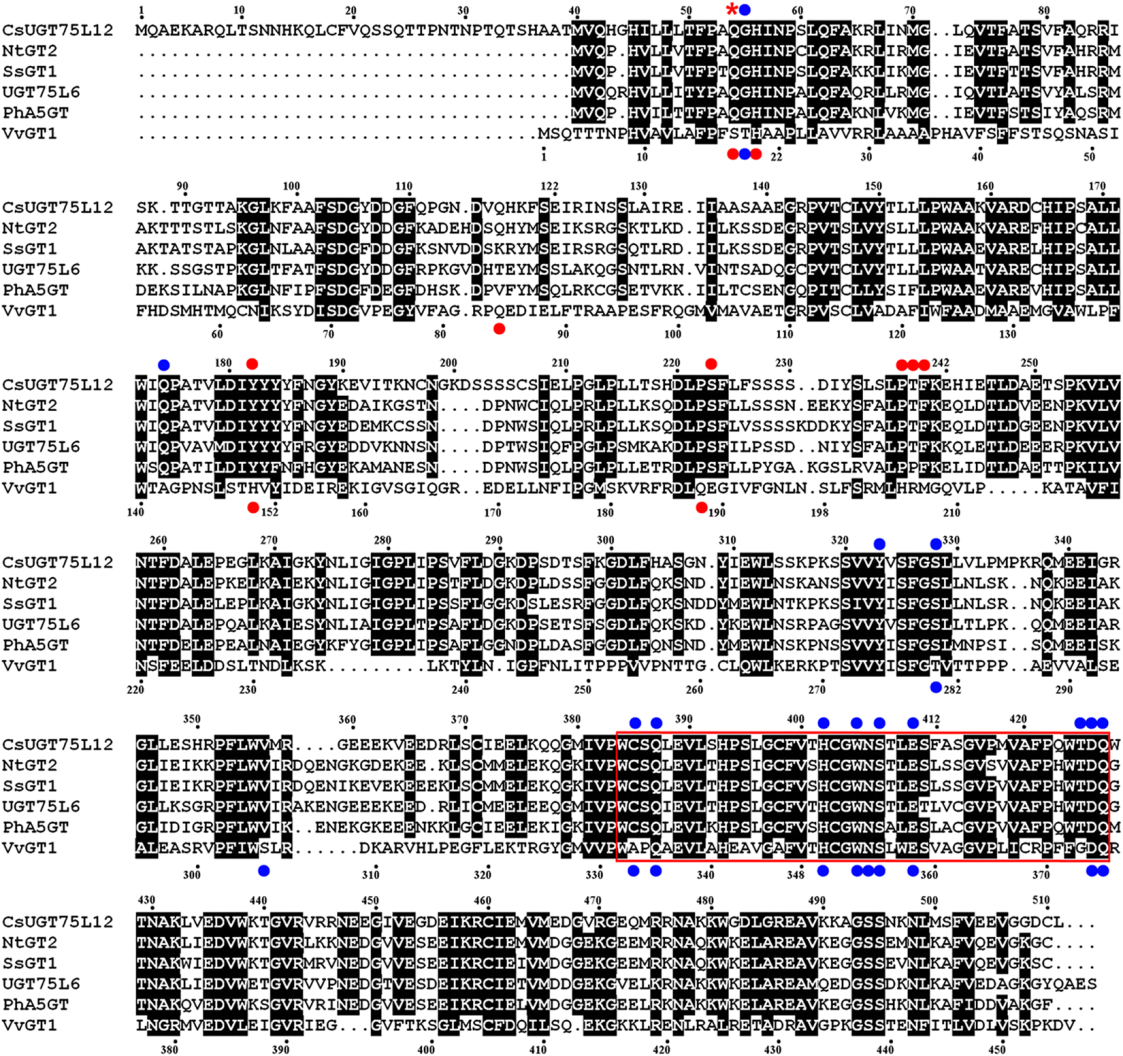
Multiple alignment of deduced amino acid sequences of CsUGT75L12 with other UGTs. The UGTs' signature PSPG motifs were enclosed in a red box. Conserved residues between the CsUGT75L12 and others UGTs were indicated with a black column. The residue denoted with a red asterisk (*) was predicted to be a sugar donor and acceptor binding site for CsUGT75L12 above the aligned sequences. Conserved residues involved in sugar donor binding sites in CsUGT75L12 (above the aligned sequences) and VvGT1 (below the aligned sequences) were designated with blue dots. Conserved residues involved in sugar acceptor binding sites in CsUGT75L12 (above the aligned sequences) and VvGT1 (below the aligned sequences) were designated with red dots.

Multiple sequence alignments of CsUGT75L12 homologous revealed the conservation in the UDP-binding domain of a PSPG box, and the divergence in the sugar acceptor binding sites in CsUGT75L12 relative to other homologous sequences in cluster II, and with VvGT1, whose crystal structure has been determined (Fig. 2). Notably, an additional N-terminal region (39 aa) was present in the amino acid sequence of CsUGT75L12, which was not present in the other homologues or in VvGT1. The homology alignment revealed that the amino acid sequence of CsUGT75L12 shared a range of 46–68% identity with NTGT2, SsGT1, UGT75L6, PtA5GT, VhA5GT, PfA5GT and ThA5GT, and only shared 27% identity with VvGT1.

A transient expression assay was performed to determine whether or not the N-terminal region (39 aa) of CsUGT75L12 protein is a signal peptide. Results indicated that both CsUGT75L12 and a truncated N-terminal (39 aa) mutant (TMIII) located to the cytoplasm and nucleus of leaf cells (Suppl Figure S2). Therefore, these data support the conclusion that the N-terminal region (39 aa) is not a functional signal peptide.

### Heterogeneous expression of CsUGT75L12 in Escherichia coli and identification of the recombinant protein

Recombinant CsUGT75L12 (rCsUGT75L12) proteins expressed in *E*. *coli* were purified by affinity chromatography using amylose resin. The molecular weight of purified rCsUGT75L12 protein was approximately 95 kDa (Suppl Figure S3a). This was consistent with the predicted molecular weight sum of maltose-binding protein (MBP) (42.5 kDa) and CsUGT75L12 (52.035 kDa). A series of enzymatic activity assays were conducted using flavonoid and phenolic acid compounds as sugar acceptors, and UDP-Glc and UDP-Gal as sugar donors. The enzymatic products obtained from the enzyme assays were identified using HPLC and UPLC-MS/MS analysis. The absorption wavelength and retention times of the products were consistent with standard compounds and data provided in published references.

**Figure 3:**
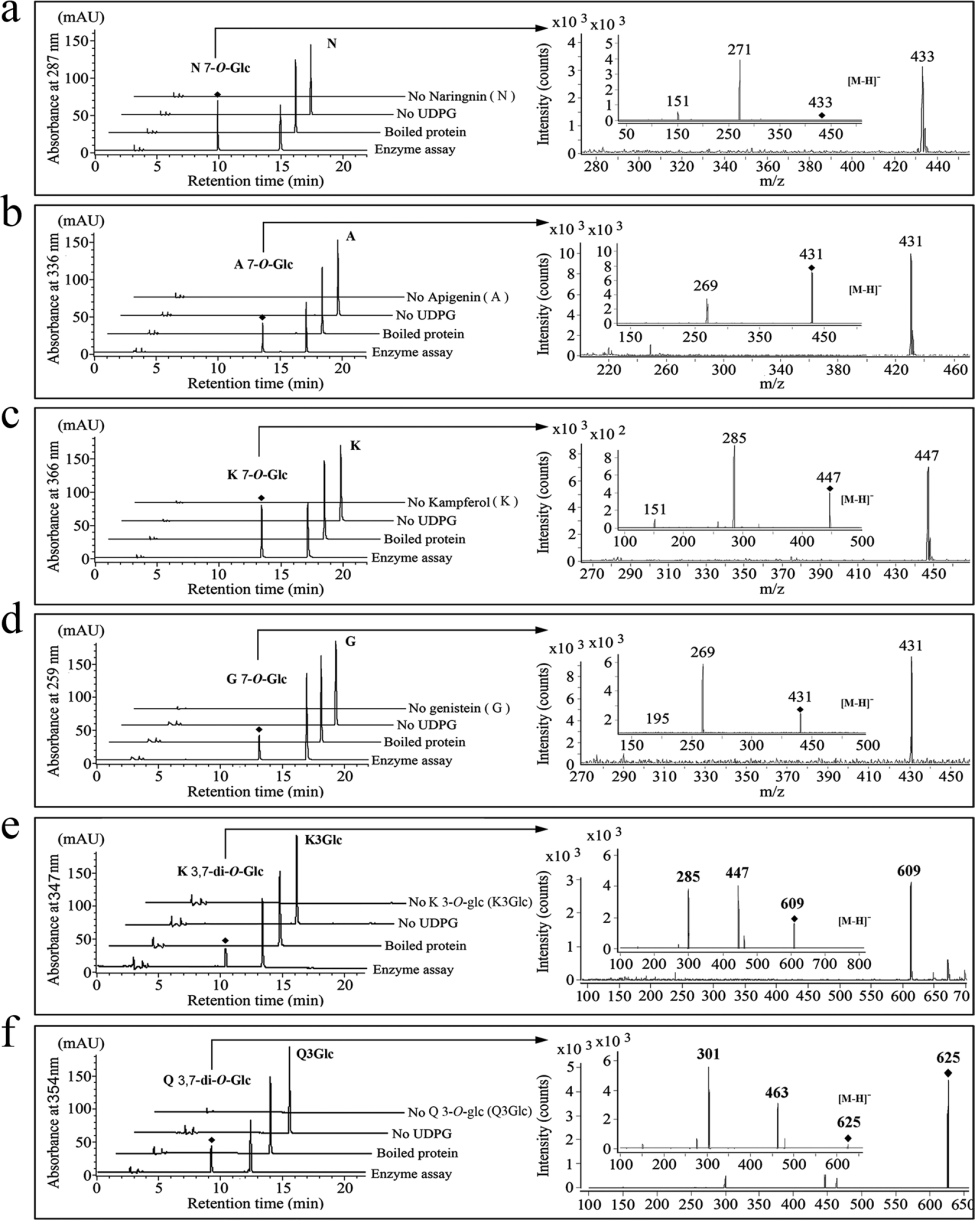
HPLC-MS/MS analysis of the enzymatic products of CsUGT75L12 protein catalysis. HPLC chromatogram (left) and MS/MS (right) analyses of the enzymatic reaction with rCsUGT75L12*in vitro*. The UDP-glucose (UDPG) and flavonoid were the sugar donor and acceptor, respectively. The substrates and corresponding products were detected by HPLC chromatogram and MS/MS. (**a**) N: naringenin and N 7-*O*-Glc: naringenin 7-*O*-glucoside; (**b**) A: apigenin and A 7-*O*-Glc: apigenin 7-*O*-glucoside; (**c**) K: kaempferol and K 7-*O*-Glu: kaempferol 7-*O*-glucoside; (**d**) G: genistein and G 7-*O*-Glc: genistein 7-*O*-glucoside. (**e**) K3Glc: kaempferol 3-*O*-glucoside and K 3,7-di-*O*-Glc: kaempferol 3-*O*,7-*O*-dioglucoside; (**f**) Q3Glc: quercetin 3-*O*-glucoside and Q 3,7-di-*O-*Glc: quercetin 3-*O*,7-*O*-dioglucoside.

The HPLC analysis indicated that rCsUGT75L12 could use several different flavonoids (naringenin, apigenin, flavonol, and genistein) and flavonoid monoglycosides (kaempferide 3-*O*-glucoside, and quercetin 3-*O*-glucoside) as sugar acceptors (Fig. 3), and both UDP-Glc and UDP-Gal could serve as sugar donors. No enzyme activity was detected in the presence of phenolic acids, anthocyanins, and catechins as sugar acceptors.

The enzymatic catalyzed products were further identified via UPLC-MS/MS analysis based on the detected deprotonated ions [M-H]^–^ and major fragments of iron (Fig. 3 and Suppl Table S2). The monoglycoside and di-glycoside were determined based on the detection of deprotonated ions [M-H]^–^ at m/z 433 (naringenin glucoside), m/z 431 (apigenin or genistein glucoside), 447 (kaempferol glucoside), 461 (kaempferide glucoside), 463 (quercetin glucoside), m/z 479 (myricetin glucoside), m/z 609 (kaempferol 3-*O*-di-galacoside/glucoside), m/z 625 (quercetin 3-*O*-di-galacoside/glucoside). The major characteristics of flavonol iron fragments were detected at m/z 285 (kaempferol), m/z 301 (quercetin), m/z 317 (myricetin), m/z 271 (naringenin), and m/z 269 (apigenin or genistein) (Suppl Table S2).

A scaled-up purification of the enzyme reaction product, kaempferol glucoside, was performed in order to confirm the position of the sugar acceptor following glucosylation catalyzed by rCsUGT75L12. The resulting purified product was identified by ^1^H NMR analysis. The ^1^H NMR spectra revealed that the chemical shifts occurring at C6 and C8 in the product, kaempferol glucoside, had transformed into a lower field (δ = 6.42 and 6.80, respectively), relative to those in the kaempferol substrate (δ = 6.19 and 6.44, respectively)(Suppl Table S3). These results further demonstrated that the glucosylation catalyzed by rCsUGT75L12 occurred at the 7-hydroxy group of the substrate flavonoid rather than at the 3-hydroxy group (Suppl Figure S4).

The UPLC-MS/MS results also indicated that no product was detected when kaempferol 7-*O*-glucoside was used as the sugar acceptor (data not shown). In contrast, kaempferol-di-glucoside and quercetin-di-glucoside were detected when kaempferol 3-*O*-glucoside and quercetin 3-*O*-glucoside were used as the sugar acceptors, respectively (Fig. 3 and Suppl Table S2). These results further demonstrated that the glucosylation catalyzed by rCsUGT75L12 took place at the 7-hydroxy group of the substrate flavonoid rather than at the 3-hydroxy group (Suppl Figure S4).

### Biochemical characterization of the recombinant enzyme

The optimum pH and temperature of rCsUGT75L12 activity were assessed for improving productivity, using kaempferol as the sugar acceptor and UDP-Glc as the sugar donor, which were determined by measuring enzyme activity under different pH (5.0–11.0) and temperature (10–65 °C) conditions. As shown in (Suppl Figure S5a,b), rCsUGT75L12 displayed the maximum glycosyltransferase activity at 40 °C in Tris-HCl buffer (pH 9.0).

The kinetic parameters of rCsUGT75L12 associated with its sugar acceptors and sugar donors were presented in Suppl Figure S6a–e. The results, based on hyperbolic Michaelis–Menten saturation curves, indicated that the corresponding *K*m values for apigenin, naringenin, kaempferol, and genistein were 4.6 μM, 68.4 μM, 7.2 μM, and 103.7 μM, respectively. In contrast, rCsUGT75L12 displayed a relative low *K*m value (233.3 μM) for the sugar donor, UDP-Glc. It was difficult to determine the kinetic parameters of rCsUGT75L12 protein toward the sugar donor UDP-Gal due to its lower catalytic activity. Overall, the results suggested that CsUGT75L12 may exhibit a higher affinity for apigenin, kaempferol, and UDP-Glc *in* *vivo* (Suppl Figure S6a,c,e).

### Expression of CsUGT75L12 in Arabidopsis thaliana

**Figure 4:**
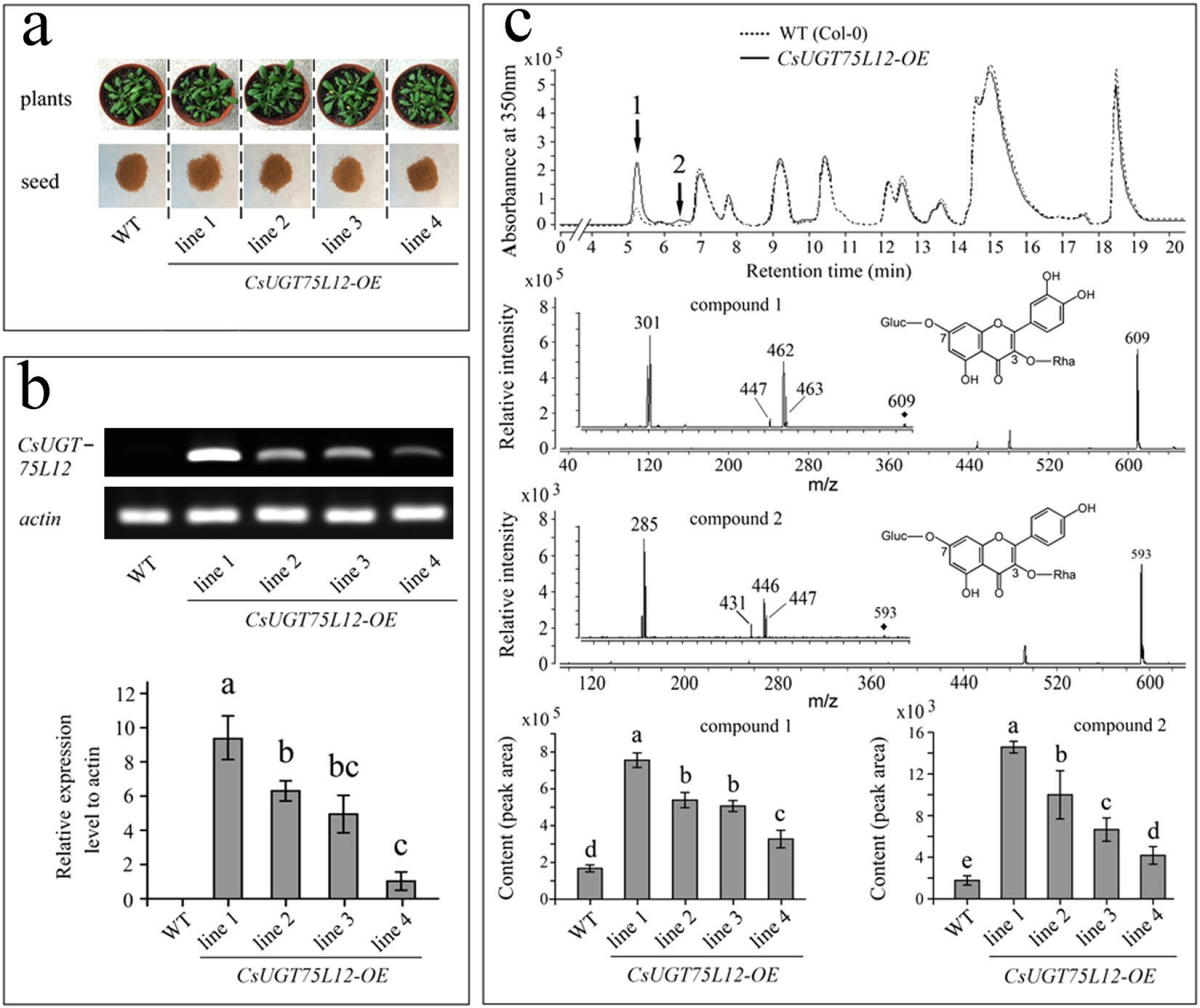
Overexpression analysis of the *CsUGT75L12* gene in Arabidopsis thaliana. The function of *CsUGT75L12* gene was verified by phenotype and metabolite variations in transgenic *A*. *thaliana*. (**a**) Phenotype of leaves and seeds of transgenic *A*. *thaliana*. (**b**) Expression level analysis of *CsUGT75L12* gene in transgenic lines. (**c**) Identification and relative content of flavonol glycosides in the seeds of different lines. Each data point was present with the average mean of three reactions ± SD. Labelled columns not connected by the same letter are significantly different at P < 0.05, based on a Tukey’s honestly significant difference test.

Independent F2 transgenic lines were screened using herbicide resistance and the presence of the transgene was confirmed by PCR analysis (Fig. 4a). Semi-quantitative RT-PCR and RT-qPCR analysis indicated that *CsUGT75L12* exhibited high levels of expression in *Arabidopsis* (Fig. 4b and Suppl Figure S7a,b). No visible phenotypic alterations were observed in the transgenic seedlings or seed coats, relative to WT seedlings and seeds (Fig. 4a). Endogenous flavonoids and their glucosides were extracted and identified from the seeds of overexpressing *CsUGT75L12* lines and WT plants. UPLC-MS/MS analysis indicated that compound 1 and compound 2 increased by 63–377% and 34–470% in seeds of the different transgenic lines, relative to seeds from WT plants (Fig. 4c). Results from compound 1 indicated that two competitive alterations had occurred, resulting in either the elimination of the first rhamnose (146 u) or alternatively, a hexose (162 u) residue to produce fragment ions at *m/z* 463 and 447, respectively. Subsequently, the remaining sugar was decomposed to give the product ion m/z 301(quercetin). Compound 2 also revealed two main product ions (*m/z* 447 and 431) which indicated that one of the sugar residues (rhamnose (146 u) or a hexose (162 u) was lost. The loss of a glucose (162 u, *m/z* 447) resulted in the presence of fragment irons for kaempferol (*m/z* 285). These data supported the premise that compound 1 was a di-*O*-glycosylated quercetin 3-*O*-rhamnoside 7-*O*-glycoside (5.205 min, m/z 609) and compound 2 was a kaempferol 3-*O*-rhamnoside 7-*O*-glycoside (6.373 min, m/z 593) (Fig. 4c). These assignments are consistent with the features of the 3,7-di-*O*-glycosylated flavonols described in previous research in *A*. *thaliana* ^34^. Collectively, the results suggested that the function of CsUGT75L12 was to transfer a glycose molecule to the C7 hydroxy group of flavonoids and form a flavonoid 7-*O*-glycoside *in planta*.

### A three-dimensional model of CsUGT75L12 protein and a molecular docking prediction

**Figure 5:**
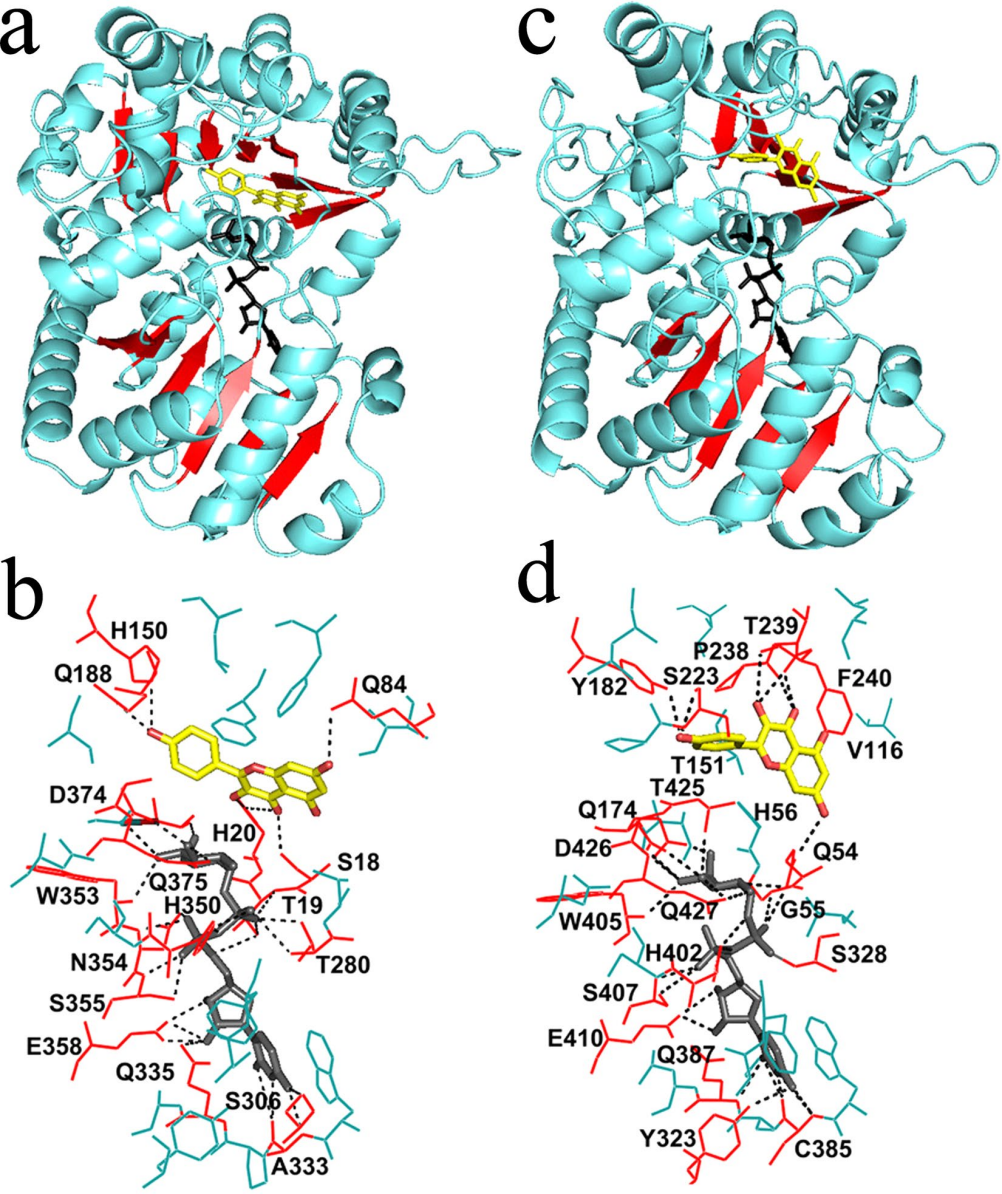
The 3D crystal modeling and molecular docking analysis of CsUGT75L12 and VvGT1 protein. (**a**,**c**) Three-dimensional rystal cartoon model of VvGT1 protein (left) and CsUGT75L12 protein (right); (**b**,**d**) Protein-ligand molecular docking analysis of VvGT1 protein (left)/CsUGT75L12 protein (right) with UDP-D-glucose (black) and kaempferol (yellow).

A three-dimensional (3D) structural model of CsUGT75L12 was constructed, using the crystal structure of VvGT1 (2c1z) as a template (Fig. 5a,b), in order to predict the involvement of key catalytic amino acids residues in glucosylation activity. Using the SWISS-MODEL website (http://swissmodel.expasy.org/), the predicted secondary structure of CsUGT75L12 exhibited 30.14% identity and 36% similarity to VvGT1. In spite of the low amino acid sequence identity, their secondary and tertiary structures are highly conserved^12^. The 39 N-terminal amino acid sequence of CsUGT75L12 was excluded in the predicted secondary structure, which was done because there was no matched template sequences for this region. The molecular docking analysis of the three-dimensional structure model (PDB file) of CsUGT75L12 was performed using the sugar donor, UDP-Glc and acceptor flavonol, kaempferol. Molegro virtual docker software^28, 35^ was used to conduct the analysis. The docking results were evaluated by using the lowest energy values. The skeleton structure model of CsUGT75L12 protein, including substrate linkage pockets and binding sites, was visualized using Python 27 software^36^.

Surprisingly, a ‘reversed’ acceptor binding mode was observed in the CsUGT75L12 skeleton structure model (Fig. 5c,d) compared to the ‘forward’ binding mode of VvGT1 (Fig. 5a,b). The 7-OH of the ‘reversed’ acceptor binding mode directly points to the Q54 residue of CsUGT75L12, which is located in the bottom of substrate pocket. Based on homology modeling, the Q54 residue was predicted to be not only a sugar acceptor binding site but also a sugar donor binding site (Fig. 5d). The H56 residue of CsUGT75L12 corresponded to the His20 residue of VvGT1, which usually was a basic residue for VvGT1 catalytic activity. Residue H56, however, is located in the side of the lower part of the substrate pocket and did not directly interact with the 7-OH of the substrate, kaempferol (Fig. 5d). Meanwhile, the 3-OH site of the substrate, kaempferol, was predicted to have a direct interaction with both residues T239 and F240, and the 4-oxygen atom of carbonyl in both P238 and T239 residues. The P238, T239 and F240 residues were located in the side of the top part of substrate pocket. The 5-OH of the kaempferol substrate was predicted to be close to the V116 residue with no direct interaction. The 4′-OH in B-ring of the substrate kaempferol was predicted to form two hydrogen bond with both Y182 and S223 residues, which are located in the other side of the top part of the substrate pocket (Fig. 5d). The overall analysis suggested that the amino acid residues Q54, Y182, S223, P238, T239, and F240 were most likely key sites for forming a ‘reversed’ acceptor binding mode for glycosylation at the 7-OH of the acceptor.

In comparison to VvGT1, the Q174 residue of CsUGT75L12, located in the sugar donor linkage pocket, was predicted to be a novel site that directly interacted with the sugar donor UDP-glucose. Most residues were found to be conservative in forming an interaction with the sugar donor, including Q387, H402, W405, S407, E410, D426, and Q427 residues of CsUGT75L12, corresponding to Q335, H350, W353, S355, E358, D374, and Q375 of VvGT1 (Figs 2 and 5d).

### Truncation of CsUGT75L12

**Figure 6:**
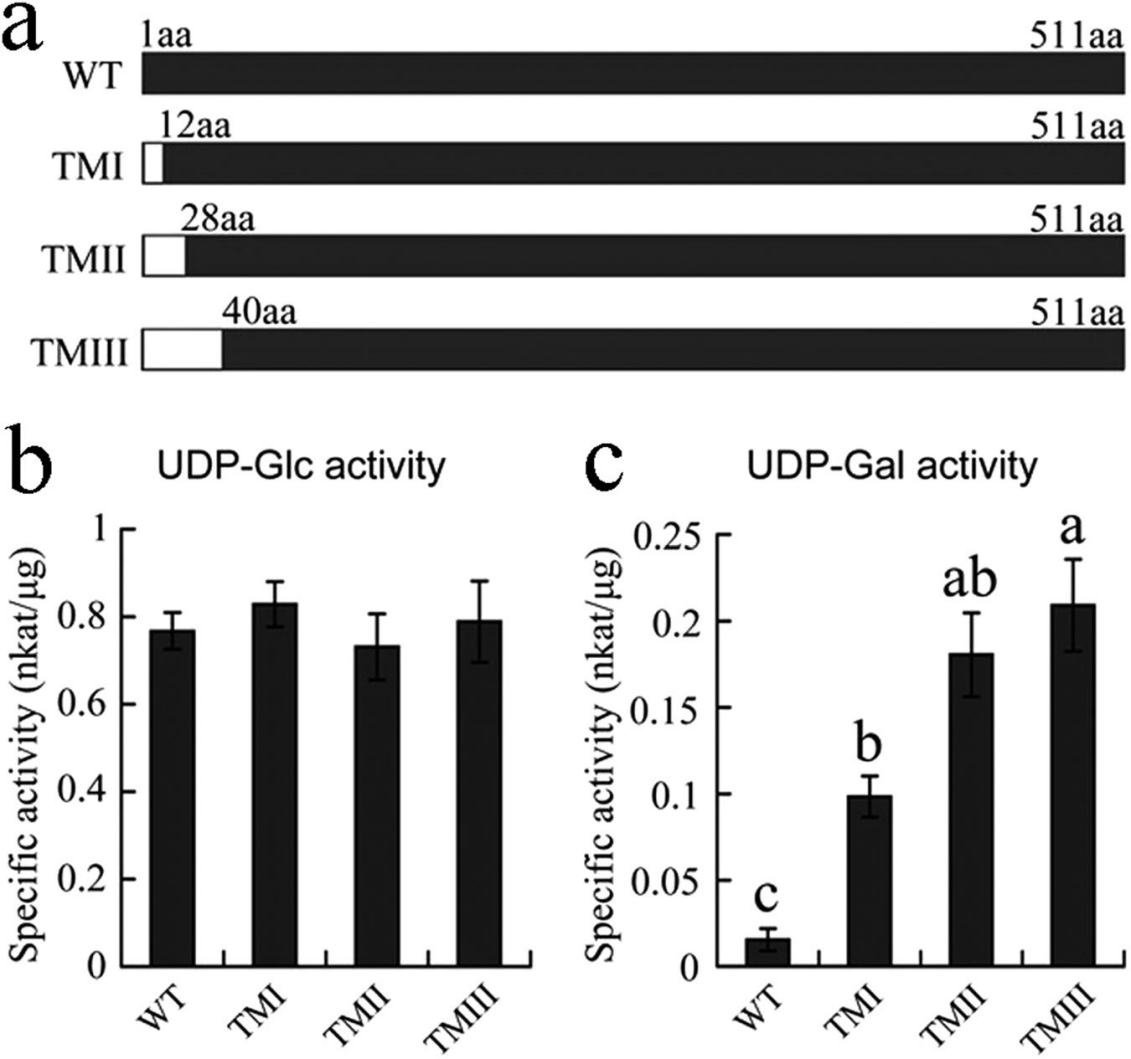
Catalytic activity analysis of CsUGT75L12 truncation mutation. (**a**) Model of the truncation position within the amino acid sequence; (**b**) Enzymatic activity analysis of truncation mutations with UDP-Glc as a sugar donor and kaempferol as an acceptor. (**c**) Enzymatic activity analysis of truncation mutations with UDP-Gal as a sugar donor and kaempferol as an acceptor. WT (nature protein 511aa); TMI (11aa truncation at the N–terminus); TMII (27aa truncation at the N–terminus), and TMIII (39aa truncation at the N–terminus). Each data point was present with the average mean of three independent trials ± SD. Labelled columns not connected by the same letter are significantly different at P < 0.05, based on a Tukey’s honestly significant difference test.

In order to assess the function of the additional N-terminal amino acids (39 aa), this portion of the CsUGT75L12 sequence was removed and the recombinant protein was expressed in *E*. *coli*. Several independent lines were created and recombinant protein was then collected, purified, and used in enzymatic assays. Results of the enzymatic assay indicated that galactosyltransferase (GalT) activity of recombinant protein in the truncated mutants (including TMI, TMII, and TMIII) was significantly higher than the full-length CsUGT75L12 protein; however little effect on glucosyltransferase (GlcT) activity was observed (Fig. 6b,c). These results suggested that the N–terminal region (39 aa) of CsUGT75L12 plays a significant role in galactosyltransferase (GalT) activity.

### Site-directed mutagenesis

In order to identify amino acid residues playing a key role in the enzymatic activity of CsUGT75L12, the individual acceptor and donor binding sites were subjected to site-directed mutagenesis. The mutated residues sites included the Q54, H56, V116, T151, Q174, Y182, S223, P238, T239, F240 and triple substitution PTF/HRM (P238H, T239R and F240M). The sites were selected based on the molecular docking model that was constructed and described in a previous section of this report. The enzymatic activity of both the full-length, recombinant CsUGT75L12 protein (approximately 95 kDa) and recombinant TMIII protein (approximately 88 kDa)(Suppl Figure S3a) were used for comparison. Kaempferol served as the sugar acceptor and both UDP-glucose and UDP-galactose served as sugar donors.

**Figure 7:**
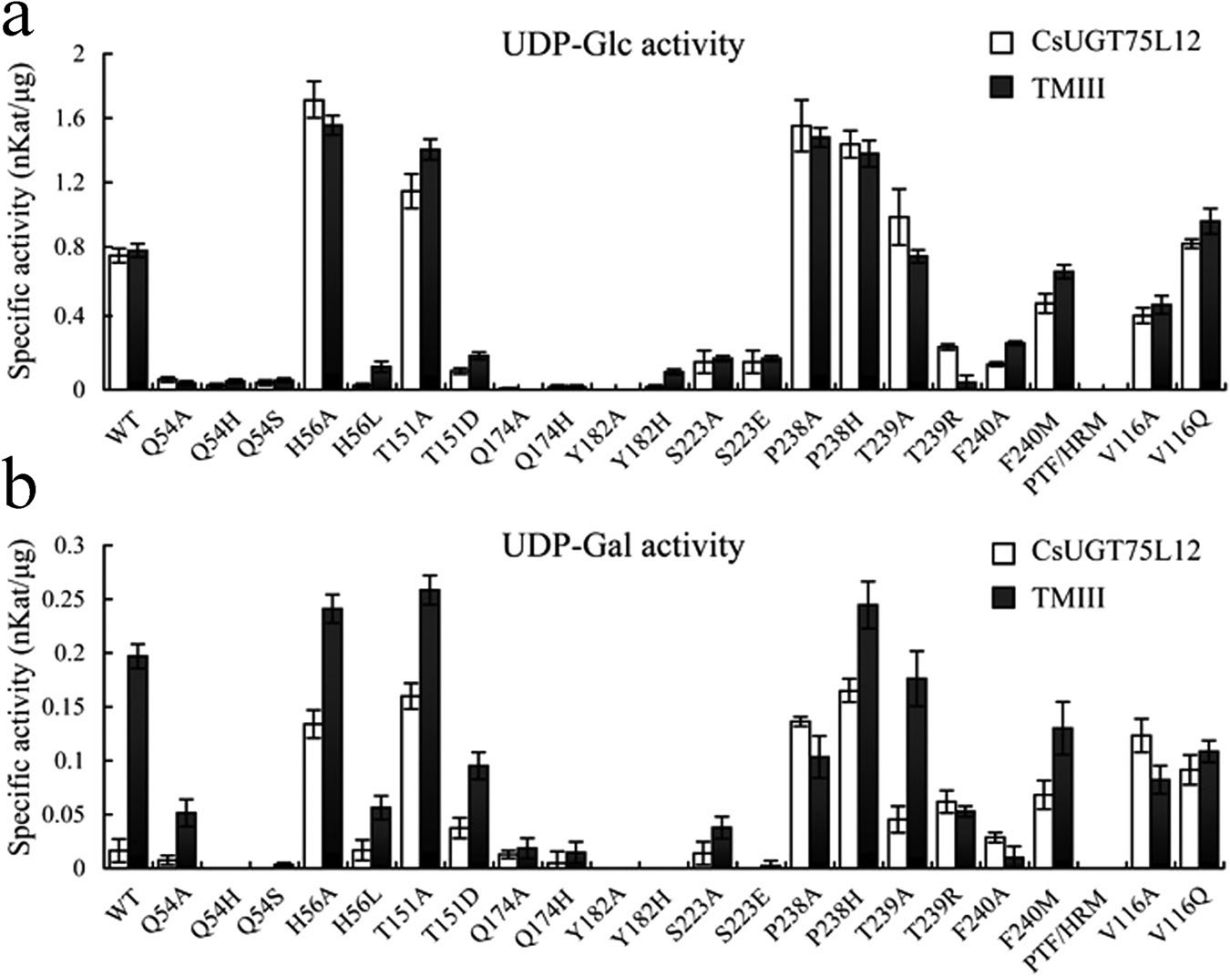
The specific activity of purified wild type and mutation proteins. (**a**) Specific activity of purified wild type and mutation proteins with UDP-Glc as sugar donor and kaempferol as acceptor. (**b**) Specific activity of purified wild type and mutation proteins with UDP-Gal as sugar donor and kaempferol as acceptor. Data were presented as the average mean of three independent trials ± SD.

**Figure 8:**
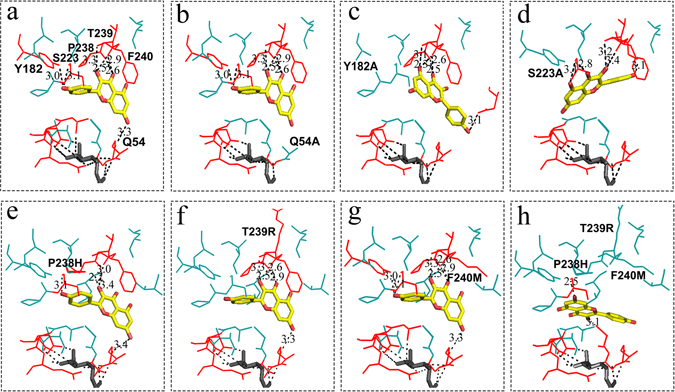
Structure models of the sugar donor and acceptor binding sites of the CsUGT75L12 and the variants. Docking molecular interaction of the sugar donor molecule UDP-Glc (dark grey) and the sugar acceptor kaempferol (yellow). The amino acid residues involved in interaction with sugar donor and acceptor were highlighted in red, and the hydrogen bonds were indicated with black dotted lines. (**a**) CsUGT75L12 (WT); (**b**) single mutation (Q54A); (**c**) single mutation (Y182A); (**d**) single mutation (S223A); (**e**) single mutation (P238H); (**f**) single mutation (T239R); (**g**) single mutation (F240M); (**h**) triple mutation (P238H, T239R and F240M).

Site-directed mutagenesis studies in VvGT1 identified several amino acid residues in the N-terminal region that played a key functional role in acceptor substrate binding and enzyme activity, including His-20 and Asp-119 of VvGT1. In the present study, replacement of His with Leu at the H56L site destroyed GlcT and GalT catalytic activity; while substitution at the H56A site increased activity (Fig. 7a,b). These results indicated that the residue at H56, corresponding to the His20 of VvGT1, was not mandatory for the catalytic activity of CsUGT75L12 protein. Substitution at the T151A site increased GlcT and GalT catalytic activity, while substitutions as the T151D site decreased catalytic activity. The question arises as to why the catalytic activity of the both CsUGT75L12 and MTIII increased when a single substitution at either H56A or T151A was made? Results of modeling simulation suggested that single substitution may render the substrate binding pocket of CsUGT75L12 more ‘open’ when compared to the wild type (Suppl Figure S8). The results of protein-ligand molecular docking analysis indicated that Q54 residue had a direct interaction with the donor and acceptor by forming two hydrogen bond (Fig. 8a), this amino acid residue appears to play a key role in enzyme activity. Indeed, substitutions at Q54A, Q54H, and Q54S resulted in weak GlcT and GalT catalytic activity in both CsUGTL12 and TMIII proteins (Fig. 7a,b).

Single substitutions at Y182H and Y182A, S223A and S223E almost completely abolished the enzymatic activity of both CsUGT75L12 and MTIII proteins (Fig. 7a,b). The molecular docking analysis indicated that the conformation of the acceptor binding mode was transposed 180 degree in the horizontal direction in the Y182A and S223A mutants compared to the structure of the native protein (Fig. 8c,d). These data suggested that the residues at the Y182 and S223 sites were responsible for the ‘reversed’ acceptor binding mode by forming hydrogen bonds with the 4′-OH group of the acceptor. The individual amino acid substitutions at sites P238A and P238H, T239A and T239R, F240A and F240M did not abolish the catalytic activity of CsUGT75L12 and MTIII proteins (Fig. 7a,b). This indicated that the function of the three residues located at P238, T239, and F240 may substitute or compensate for each other. Interestingly, the molecular docking analysis supported this premise that the triple substitution of the amino acids located at P238H, T239R, and F240M would change the configuration of the acceptor binding mode from ‘reversed’ to ‘forward’ (Fig. 8h). Indeed, the catalytic activities of both CsUGT75L12 and MTIII protein could be abolished when the amino acids at all three sites, (P238H, T239R and F240M) were all substituted together (Fig. 7a,b).

Individual substitutions at V116A and V116Q did not abolish the catalytic activity of CsUGT75L1 and MTIII protein, which indicated that amino acid located at the V116 site was functionally unimportant for catalytic activity. The results of the enzymatic activity assay for the mutated proteins demonstrated that a single substitution at either Q174A or Q174H essentially abolished both the GlcT and GalT activity of CsUGT75L1 and MTIII proteins (Fig. 7a,b).

## Discussion

### The function and significance of the *CsUGT75L12* gene in tea plants

A phylogenetic analysis of flavonoid glycosyltransferases (UFGTs) in plants revealed that they could be grouped into five functional clusters. Clusters I, II, and III (included IIIa and IIIb) are predicted to be UFGTs with different regiospecificity of the sugar acceptor. The UFGTs include flavonoid 3-*O*-glycosyltransferases, 5-*O*-glycosyltransferases, and 7-*O*-glycosyltransferases^15, 30, 31, 37, 38^. Members of cluster IV are characterized by branch-forming glycosyltransferases, such as the 1,6-rhamnosyltransferase from citrus fruit, which was found to catalyze branched-chain rhamnosylation of flavonoids glucosylated at positions 3 or 7 hydroxy group^39–41^.

Although the phylogenetic analysis can provide information that is able to partially predict the biochemical activities of plant UGTs, some of the genes which were classified in the same cluster exhibited different functional glycosylation sites in previous research^11, 32, 33^ and our current study. For instance, even though ThA5GT, VhA5GT, PfA5GT, IhA5GT, SsGT1, NTGT2, and UGT75L6 group in Cluster II (which was predicted to exhibit anthocyanin 5-*O*-glycosyltransferases activity); SsGT1 and NTGT2 were functionally proven to display flavonoid 7-*O*-glucosyltransferases activity^11, 32^, additionally, UGT75L6 from *Gardenia jasminoides* preferentially glucosylates the carboxyl group of crocetin to form a crocetin glucose ester^33^.

In the present study, the *CsUGT75L12* gene was cloned from tea plants. Although CsUGT75L12 was grouped into Cluster II based on a phylogenetic analysis (Fig. 1), recombinant CsUGT75L12 protein expressed in *E*. *coli* exhibited glucosylation acitivity towards multiple phenolic substrates at the 7-OH position with the exception of anthocyanin, catechins, and phenolic acid (Fig. 3 and Suppl Table S2). The accumulation of quercetin 3-*O*-rhamnopyranoside-7-*O*-glucopyranoside and kaempferol 3-*O*-rhamnopyranoside-7-*O*-glucopyranoside were significantly promoted, relative to WT plants, in transgenic plants of *Arabidopsis thaliana* overexpressing *CsUGT75L12* (Fig. 4c). Based on these evidence of provided in the above-mentioned *in vitro* and in *vivo* experiments, CsUGT75L12 was confirmed to be a flavonoid 7-*O*-glycosyltransferase.

### Crucial amino acid residues or regions involved in the regiospecificity of glycosylation

Recent studies have revealed that the loop regions, domains and segments, and amino acid residues of UFGTs associated with its secondary structure are functionally important for the enzymes to exhibit glucosyltransferase catalysis activity^23, 24, 37, 42^. This evidence has been derived from the elucidation of crystal structure, and site-directed mutagenesis studies^15, 43–45^.

The sugar acceptor pocket in UFGTs is almost entirely formed by N-terminal residues, most of which are located in loopN1, loopN2, Na3, loopN4, Na5, loopC1, C5 loop^12, 13, 45, 46^. Site-directed mutagenesis can be used to demonstrate the importance of specific amino acid residues in the catalytic activity of a protein. In our study, several amino acid as key residues that are necessary for substrate binding and enzyme activity. These data were in accordance to a previous report. For His20 and Asp119 in VvGT1^25^, site-directed mutagenesis indicated that the His residue was essential for catalytic activity, and that the Asp residue served as a partner, assisting His20 by initializing or activating the reaction through electron transfer^16^. Similarly, these residues at H19 and D117 of UGT72B1 in *Arabidopsis thaliana* ^22^, and at H21 and D125 of UGT85H2 in *Medicago truncatula* were also important for enzymatic activity^26^. Unexpectedly, we found that the substitutions made at the H56A and T151A sites of both rCsUGTL12 and TMIII protein displayed high glucosyltransferase and galactosyltransferase catalytic activity. However, the catalytic activity of the H56L and T151D mutants, relative to the WT protein, was abolished (Fig. 7a,b). The results indicated that residues H56 and T151, corresponding to the basic active residues H20 and D119 of VvGT1, were not irreplaceable for UGT75L12 catalytic activity. A similar phenomenon has been reported in other plants. For example, it was also determined that His15 and Asp125 in GmIF7GT from *Glycine max* were also not important for its catalytic activity^47^.

In the present study, the predicted secondary structure of CsUGT75L12 indicated that the Q54 amino acid residue may be the catalytic basic residue site, and that residues at Y182, S223, P238, T239, and F240 are potential key sites necessary for forming a ‘reversed’ acceptor binding mode for the regiospecificity glycosylation at the 7-OH of an acceptor (Fig. 8b–h). This premise is obtained based on the molecular docking analysis of three-dimensional (3D) structural model of CsUGT75L12 (Figs 5d and 8a–h). Indeed, all of the proteins mutated at sites Q54A, Q54H, and Q54S displayed weak glucosyltransferase and galactosyltransferase catalytic activity in both rCsUGTL12 and the truncated, TMIII protein, which suggested that the amino acid residue at Q54 may be more important than the H56 residue for the catalytic activity of the CsUGTL12 protein.

Based on molecular docking model analysis and the site directed mutagenesis assays, the functions of three residues located at P238, T239, and F240 were found to be interesting, as they could be substitued by each other to maintain a ‘reversed’ acceptor binding mode by forming stable hydrogen bonds with the 3-OH and 4-carbanyl group of the kaempferol substrate. The molecular docking analysis indicated that substitution of any one of the residues at P238, T239, or F240 did not change the ‘reversed’ acceptor binding mode (Fig. 8e–g). Only a triple substitution of all three residues at P238H, T239R, and F240M had an obvious effect on the conformation which shifted the ‘reversed’ acceptor binding mode to a ‘forward’ mode (Fig. 8h). The site-directed mutagenesis studies revealed that a single substitution at P238A, P238H, T239A, T239R, F240A, and F240M did not abolish the catalytic activity of CsUGT75L12 or the truncated, MTIII protein(Fig. 7a,b). However, a triple amino acid substitution at P238H, T239R, and F240M of CsUGT75L12 and MTIII protein, completely abolished catalytic activity (Fig. 7a,b).

Few studies have reported any functional significance to the N-terminal region for recognition of a sugar donor. In comparison to the others plant UGTs, CsUGT75L12 has an additional 39-amino acid segment at the N-terminal end (Fig. 2). Interestingly, the mutated protein in which the N-terminal 39-residue segment was deleted to display higher galactosyltransferase activity than the wild type protein (Fig. 6c), but the truncation did not have any effect on glucosyltransferase activity (Fig. 6b). Therefore, it could be speculated that the N-terminal 39-residue segment of CsUGT75L12 may be related to the sugar donor recognition specificity.

With respect to the sugar donor linkage pocket, the amino acid residue located at Q174 of CsUGT75L12 exhibited novel activity compared to the same site in VvGT1. The protein-ligand molecular docking analysis indicated that the residue at Q174 could directly interact with the UDP-glucose sugar donor (Fig. 5d). A single substitution at Q174A or Q174H essentially abolished both the GlcT and GalT activity of CsUGT75L1 and MTIII proteins (Fig. 7a,b). These results indicated that the residue at Q174 played an important role in the 7-*O*-glycosyltransferase activity of CsUGT75L1.

## Methods

### Plant material

Different aged tissues (including seedlings, young shoots, and tender roots) of tea plants (*Camellia sinensis* var. *sinensis* cv. ‘Nongkangzao’) growing at the experimental tea garden of Anhui Agricultural University (Anhui, China) were collected during early spring and used in the present study. All of the samples were immediately frozen in liquid nitrogen and stored at −80 °C prior to further use.

### Chemicals

Kaempferide and apigenin were purchased from Chengdu Must Bio-technology Co., Ltd (Chengdu, China). Naringenin, Gallic acid, benzoic acid, caffeic acid, ferulic acid, sinapic acid, cyanidin, catechins, UDP-glucose, and UDP-galactose were obtained from Sigma (St Louis, MO, USA). Quercetin 3-*O-* galactoside, kaempferol 3-*O-* galactoside, kaempferol 3-*O*-glucoside, kaempferol 7-*O*-glucoside, myricetin 3-*O*-galactoside, quercetin, kaempferol, myricetin and genistein were purchased from Shanghai Winherb Medical Technology Co., Ltd (Shanghai, China).

### RNA isolation, cDNA Cloning, and Sequence analysis

Total RNA was isolated and extracted from different samples using RNAiso-mate for Plant Tissue (Takara, DaLian, China; Code: D325A) and RNAiso Plus (Takara, DaLian, China; Code: D9108B) according to the manufacturer’s instructions. cDNA was synthesized by reverse transcription from the total RNA using PrimeScript RT Master Mix (Takara, DaLian, China;Cat: RR036A). The 3′-cDNA and 5′-cDNA fragments of candidate genes were synthesized using a SMARTer™ RACE cDNA Amplification Kit (Clontech, USA; Cat. Nos. 634923 and 634924). A sequence for the full length open reading frame (ORF) was obtained by assembling the obtained 5′ and 3′ sequences. Subsequently, the ORF sequence was amplified using Phusion® High-Fidelity DNA Polymerase (New England Biolabs, USA). The amplified product was designated as ‘*CsUGT75L12*’, using the naming regulations set forth by the UGT Nomenclature Committee, and submitted to NCBI, (GenBank TM accession number KP682364). All of the primers sequences that were used in the study are listed in Suppl Table S1.

Sequence alignments were conducted using Dnaman 7 software (Lynnon, Canada). The phylogenetic tree was generated by the neighbor-joining method within the MEGA 5.0 program using 1,000 bootstrap replications.

### Subcellular localization

Full-length coding sequences without a stop codon of *CsUGT75L12* and *TMIII* (39aa truncation at the N–terminus) were cloned into the entry vector pDONR207 using a Gateway BP Clonase Enzyme mix according to the manufacturer’s instructions (Invitrogen, USA). Positive clones of entry pDONR207-CsUGT75L12 were selected for on gentamycin plates and further validated by PCR. Entry vectors were then transferred into the Gateway plant transformation destination vector, pGWB5, to construct C-terminal GFP fusion proteins using Gateway LR Clonase (Invitrogen, USA).

Recombinant colonies pGWB5-CsUGT75L12-GFP, pGWB5-TMIII-GFP, and the control pGWB5-GFP-GFP vectors were selected for on kanamycin plates and further validated by PCR. The localization constructs were electroporated into *A*. *tumefaciens* EHA105 cells and confirmed by PCR analysis. For transient expression analyses, the three correct constructs were injected into *Nicotiana benthamiana* leaves. The infiltrated leaves were cultivated in a greenhouse for 48 h, and subsequently examined under a Olympus FV1000 confocal microscope (Olympus, Tokyo, Japan). The detection wavelengths chosen were 497–531 nm for green fluorescence, 610–640 nm for red fluorescence, and 660–680 nm for chlorophyll autofluorescence. Photomicrographs were recorded and processed using Olympus Fluoview Ver.3.0 Viewer software.

### Heterologous expression in Escherichia coli and recombinant protein purification

Full-length ORF sequences of *CsUGT75L12* were obtained by end-to-end PCR using gene-specific primers (Suppl Table S1) and subcloned into the expression vector, pMAL-c2X (New England Biolabs, MA, USA). The identity of the cloned gene was confirmed using the sequencing primers: pMAL-C2X-F5′- TGCGTACTGCGGTGATCAAC-3′ and pMAL-C2X-R 5′-CTGCAAGGCGATTAAGTTGG-3′(http://www.lifetechnologies.com/). The recombinant pMAL-c2X-CsUGT75L12 was transformed into *Escherichia coli* Novablue (DE3) Competent Cells (Novagen, Schwalbach, Germany). The expression strain was grown at 37 °C in 200 mL Luria-Bertani medium containing 100 μg·ml^−1^ ampicillin and 2 g·L^−1^ glucose. Later, 0.3 mM IPTG was added to the medium when the OD_600_ reached 0.4 ~ 0.6. This culture was incubated at 37 °C for 24 h. The cells were harvested by centrifugation and stored at –20 °C overnight. Recombinant proteins were purified by affinity chromatography using amylase resin (New England Biolabs, MA, USA). For affinity chromatography, the solvent system contained two column buffers (A and B). Specifically, column buffer A (20 mM Tris-HCl, 200 mM NaCl, 1 mM DTT, and 1 mM EDTA, pH 7.4) was used to wash out unbound non-recombinant proteins and Column buffer B (column buffer A with the addition of 10 mM maltose) was used to elute the bound fusion protein from the resin. The identity of the obtained protein fraction was confirmed by electrophoresis on a 12% SDS polyacrylamide gel stained with Coomassie brilliant blue. The purified recombinant protein was used in an *in vitro* activity assay.

### Enzymatic assays and analysis of kinetic parameters

In order to analyze the activity of rCsCsUGT75L12, a reaction mixture (50 μL total volume) was made containing 100 mM Tris-HCl (pH 7.5), 2.5 mM UDP-glucose (UDP-Glc) or UDP-galactose (UDP-Gal) as the sugar donor, 200 μM of potential phenolic acid substrates (including gallic acid, benzoic acid, caffeic acid, ferulic acid, and sinapic acid), flavonoids (including quercetin, kaempferol, myricetin, naringenin, apigenin, genistein, catechin, and cyanidin chloride), and monoflavonoid glycosides (quercetin 3-*O*-glucoside, kaempferol 3-*O*-glucoside, kaempferol 7-*O*-glucoside) as the sugar acceptors, and 15 μg of purified recombinant protein. Each reaction mixture was incubated at 30 °C for 30 min and the reactions were terminated with the addition of 50 μl of methanol.

The optimal pH for rCsUGT75L12 activity was determined using a variety of pH buffer solutions, including 100 mM citric acid citrate buffer 1 (pH 4.0 to 6.0), 100 mM Tris-HCl buffer 2 (pH 6.5 to 8.5), 100 mM phosphate buffer 3 (pH 6.0 to 9.0), and 100 mM Na_2_CO_3_/NaHCO_3_ buffer 4 (pH 9.0 to 11.0). In order to determine the optimal temperature for rCsUGT75L12, the reaction mixture was incubated at a series of temperatures (10 °C, 20 °C, and 30 °C-65 °C with 5 °C increments) in 100 mM Tris-HCl buffer (pH 9.0) for 30 min. All of the reaction mixtures were supplemented with 0.1% (v/v) β-mercaptoethanol and the reactions were repeated in triplicate, along with reactions without protein extracts as controls. The glycoconjugate products were centrifuged and stored at −20 °C prior to HPLC and UPLC-MS/MS analysis.

The kinetic parameters of sugar acceptors for rCsUGT75L12 were determined using 2.5 mM UDP-Glc as the sugar donor, and 0~400 μM of flavonoid (kaempferol, apigenin, genistein, or naringenin) as the sugar acceptors. For the kinetic parameters of the sugar donor, rCsUGT75L12 was tested using 250 μM kaempferol as the acceptor and 0~2 mM of UDP-Glc as the sugar donor. Each reaction mixture was incubated at 30 °C with 100 mM Tris-HCl buffer (pH 7.5) for 10 min, and the reactions were terminated by adding 50 μl methanol. The product Uridine 5′-diphosphate (UDP) was used to quantify the kinetic parameters illustrated by F. Khater^48^ and in our previous study^5^. Calibration graphs were obtained by injecting standard UDP in the range of 20~500 μM. A capillary electrophoresis (CE) system using P/ACE MDQ (Beckman, USA) equipped with a diode array detector was used in the analysis.

### Identification of flavonoid glycoconjugate products

HPLC was used to identify the presence of glyconjugate products. The HPLC protocol utilized a C_18_ column (150 × 4.6 mm i.d.: Luna® 5 µm C18(2), Phenomenex, Torrance, CA) with an LC-10Avp system (Shimadzu, Kyoto, Japan), and a detection wavelength of 190~700 nm. The mobile phase consisted of 1% acetic acid in water (eluent A) and 100% acetonitrile (eluent B), with a flow rate of 1.0 mL/min. The elution program was executed as follows: 10–25% B for 0–5 min, 25–50%B for 5–10 min, 50–80%B for 10–17 min, 80–30% for 17–20 min, 30–10% for 20–21 min and termination at the 22 min. The Ultra Performance Liquid Chromatography (UPLC)-MS/MS system was utilized as previously described^49^.

In order to identify the glycosylation position of substrates, the kaempferol glucoside products were purified by Silica gel column chromatography and collected. The samples were evaporated and freeze-dried and dissolved in deuterated methanol. The NMR spectra of the kaempferol and its glucoside were acquired on a Bruker AVANCE AV 400-MHz NMR spectrometer at 22 °C. The data were processed and analyzed using MestReNova v. 5.2.5 software.

### Heterologous expression in Arabidopsis thaliana and flavonoid analysis

The open reading frame (ORF) of the *CsUGT75L12* gene was shuttled into the pCB2004 vector carrying the 35 S promoter using the Gateway LR reaction (Invitrogen, USA). The correct recombinant pCB2004-CsUGT75L12 were transformed into wild-type (WT) plants of *Arabidopsis thaliana* ecotype Columbia-0 (Col-0) by *Agrobacterium tumefaciens*-mediated transformation, using strain C58C1. The primers used for measuring *CsUGT75L12* gene expression were confirmed to be gene-specific and are listed in Suppl Table S1. Gene expression, relative to a control group (WT), was measured in overexpression *CsUGT75L12* lines and the resultant data were expressed as the mean of three replicates. Data was normalized against the expression value of the housekeeping gene, *Actin* and the relative expression values were calculated by the 2-ΔΔCt method^50^.

Total flavonoids were extracted from *Arabidopsis* seeds according to a previously described method^34^. The level of phenolic compounds in transgenic *CsUGT75L12* and WT plants was determined by the signal intensity (peak area) of a corresponding product ion by the MRM mode of UPLC-MS/MS. The elution program on the HPLC system was utilized as previously described^49^. Relative quantification was based on the area of major MS signals (M-H)^+^.

### Homology modeling, truncation assessment, and site-directed mutagenesis

The crystal structure of VvGT1 (PDB code 2c1z) was used to construct a homology model of CsUGT75L12 using the SWISS-MODEL server (http://swissmodel.expasy.org/interactive). The molecular interaction docking analysis of CsUGT75L12 was performed with UDP-Glc as the sugar donor and flavonol (kaempferol) as the acceptor, using Molegro virtual docker software^35^. The result of docking data were evaluated using lowest energy values. The resulting model skeleton structure of the CsUGT75L12 protein, including substrate linkage pockets and binding sites, could be observed and analyzed utilizing Python 27 software^36^.

The mutagenesis of TMI (11aa truncation at the N–terminus of CsUGT75L12), TMII (27aa truncation at the N–terminus of CsUGT75L12) and TMIII (39aa truncation at the N–terminus of CsUGT75L12 encodes a putative 46,86 kDa protein) were obtained by end-to-end PCR. A series of site-directed mutations were generated using a site-directed mutagenesis kit (Biomed, Beijing, China) with pMAL-c2X-CsUGT75L12 and pMAL-c2X-TMIII plasmids as the templates. The recombinant PCR amplifications were performed utilizing the following conditions: 98 °C for 2 min, 25 cycles at 98 °C for 10 s, 58 °C for 30 s, 72 °C for 4.5 min, and a final extension at 72 °C for 5 min. Mutations were verified by sequencing and the specific mutagenesis oligonucleotide primers are listed in (Suppl Table S1). Purified mutant proteins were obtained using the same procedures described above for native proteins. The enzyme assays of the mutated, recombinant proteins were performed under the same conditions described above for native proteins.

## Electronic supplementary material

Supplementary Information
