# Supplementary material for: Identification of a Flavonoid Glucosyltransferase Involved in 7-OH Site Glycosylation in Tea plants (Camellia sinensis)
Source: Sci Rep. 2017 Jul 19;7:5926. doi: 10.1038/s41598-017-06453-z (PMC5517534; doi:10.1038/s41598-017-06453-z)
Supplement: Supplementary file 1 — Supplementary Information [file 41598_2017_6453_MOESM1_ESM.doc]

**Supplementary Information**

Supplementary Data Figures and Tables

**Identification of a Flavonoid Glucosyltransferase Involved in 7-OH Site Glycosylation in Tea plants (*Camellia sinensis*)**

Xinlong Dai ^1, 2†^, Juhua Zhuang ^1†^, Yingling Wu^1^, Peiqiang Wang^1^, Guifu Zhao^1^, Yajun Liu^2^, Xiaolan Jiang^1^, Liping Gao^2*^and Tao Xia^1*^

**TableS 1.**The specific primers used in this study.

| **purpose** | **Gene names** | **primer name** | **primer sequence (5'-3')** |
| --- | --- | --- | --- |
| **cloning** | CsUGT75L12 | 3′-outer | GTCTTTTGGAAGCCTATTAGTGTTGCC |
|  |  | 3′-inner | GGAGGAGATTGGTCGTGGTTTACTGG |
|  |  | 5′-outer | GCTAGCGGCGATGATTTCTCTGATGGCAAG |
|  |  | 5′-inner | CTGGTTGGAACCCATCATCGTAGCCGTCG |
|  |  | End-to-end F | ATGCAAGCGGAGAAAGCTAGGC |
|  |  | End-to-end R | CTATAAGCAATCTCCTCCAACC |
|  | TMIII | End-to-end F | ATGGTGCAGCACGGCCACATCCTC |
|  |  | End-to-end R | CTATAAGCAATCTCCTCCAACC |
| **Real time PCR** | GADPH | qRT-PCR-F | TTGGCATCGTTGAGGGTCT |
|  |  | qRT-PCR-R | CAGTGGGAACACGGAAAGC |
|  | CsUGT75L12 | qRT-PCR-F | AAGCCTATTAGTGTTGCCAATGCCA |
|  |  | qRT-PCR-R | GGCACTATCATCCCTTGTTGCTTCA |
|  | Actin 2 | qRT-PCR-F | GCTGCTGGAATCCACGAGACAA |
|  |  | qRT-PCR-R | ATCCACATCTGCTGGAATGTGC |
| **pMAL-c2X cloning for fusion protein expression** | WT | CsUGT75L12-pMAL-F | TCTAGAATGCAAGCGGAGAAAGCTAGGC |
|  |  | CsUGT75L12-pMAL-R | CTGCAGCTATAAGCAATCTCCTCCAACC |
|  | TMI | TMI-pMAL-F | TCTAGAATGAACAACCACAAACAACTCTG |
|  |  | TMI-pMAL-R | CTGCAGCTATAAGCAATCTCCTCCAACC |
|  | TMII | TMII-pMAL-F | TCTAGAATGAATACCAACCCCACACAAAC |
|  |  | TMII-pMAL-R | CTGCAGCTATAAGCAATCTCCTCCAACC |
|  | TMIII | TMIII-pMAL-F | TCTAGAATGGTGCAGCACGGCCACATCCTC |
|  |  | TMIII-pMAL-R | CTGCAGCTATAAGCAATCTCCTCCAACC |
| **Expression in Arabidopsis thaliana** | CsUGT75L12 | CsUGT75L12-BP-F | GGGGACAAGTTTGTACAAAAAAGCAGGCTATGCAAGCGGAGAAAGCTAGG |
|  |  | CsUGT75L12-BP-R | GGGGACCACTTTGTACAAGAAAGCTGGGTCTATAAGCAATCTCCTCCAACC |
| **transient expression in Nicotiana benthamiana** | CsUGT75L12 | CsUGT75L12-GFP-F | GGGGACAAGTTTGTACAAAAAAGCAGGCTATGCAAGCGGAGAAAGCTAGG |
|  |  | CsUGT75L12-GFP-R | GGGGACCACTTTGTACAAGAAAGCTGGGTGTAAGCAATCTCCTCCAACC |
|  | TMIII | TMIII-GFP-F | GGGGACAAGTTTGTACAAAAAAGCAGGCTATGGTGCAGCACGGCCACATC |
|  |  | TMIII-GFP-R | GGGGACCACTTTGTACAAGAAAGCTGGGTGTAAGCAATCTCCTCCAACC |

**TableS 1.** cont.

| **purpose** | **Gene names** | **primer name** | **primer sequence (5'-3')** |
| --- | --- | --- | --- |
| **Site-Directed Mutagenesis** | CsUGT75L12  and  TMIII | Q54A-F | CTGACTTTTCCGGCAGCAGGCCACATCAACCC |
|  |  | Q54A-R | GGGTTGATGTGGCCTGCTGCCGGAAAAGTCAG |
|  |  | Q54H-F | GACTTTTCCGGCACACGGCCACATCAACCCT |
|  |  | Q54H-R | AGGGTTGATGTGGCCGTGTGCCGGAAAAGTC |
|  |  | Q54S-F | CTGACTTTTCCGGCATCAGGCCACATCAACCC |
|  |  | Q54S-R | GGGTTGATGTGGCCTGATGCCGGAAAAGTCAG |
|  |  | H56L-F | CCGGCACAAGGCCTCATCAACCCTTCTCTC |
|  |  | H56L-R | GAGAGAAGGGTTGATGAGGCCTTGTGCCGG |
|  |  | H56A-F | CCGGCACAAGGCGCCATCAACCCTTCTCTCC |
|  |  | H56A-R | GGAGAGAAGGGTTGATGGCGCCTTGTGCCGG |
|  |  | V116A-F | CCAACCAGGAAACGAT GCACAACACAAGTTCTCCG |
|  |  | V116A-R | CGGAGAACTTGTGTTGTGCATCGTTTCCTGGTTGG |
|  |  | V116Q-F | CCAACCAGGAAACGATCAACAACACAAGTTCTCC |
|  |  | V116Q-R | GGAGAACTTGTGTTGTTGATCGTTTCCTGGTTGG |
|  |  | T151D-F | ACCTGCTTGGTCTACGATCTCCTCCTCCCTTG |
|  |  | T151D-R | CAAGGGAGGAGGAGATCGTAGACCAAGCAGGT |
|  |  | T151A-F | ACCTGCTTGGTCTACGCTCTCCTCCTCCCTTG |
|  |  | T151A-R | CAAGGGAGGAGGAGAGCGTAGACCAAGCAGGT |
|  |  | Q174A-F | CAGCTCTTCTCTGGATTGCACCTGCCACAGTTTTAG |
|  |  | Q174A-R | CTAAAACTGTGGCAGGTGCAATCCAGAGAAGAGCTG |
|  |  | Q174H-F | CTCTTCTCTGGATTCATCCTGCCACAGTTTTAG |
|  |  | Q174H-R | CTAAAACTGTGGCAGGATGAATCCAGAGAAGAG |
|  |  | Y182A-F | CCACAGTTTTAGACATAGCCTACTATTACTTCAATG |
|  |  | Y182A-R | CATTGAAGTAATAGTAGGCTATGTCTAAAACTGTGG |
|  |  | Y182H-F | CACAGTTTTAGACATACACTACTATTACTTCAATG |
|  |  | Y182H-R | CATTGAAGTAATAGTAGTGTATGTCTAAAACTGTG |
|  |  | S223A-F | GCCATGACCTTCCTGCCTTTCTATTCTCTTC |
|  |  | S223A-R | GAAGAGAATAGAAAGGCAGGAAGGTCATGGC |
|  |  | S223E-F | CACTAGCCATGACCTTCCTGAGTTTCTATTCTCTTCAAGCTC |
|  |  | S223E-R | GAGCTTGAAGAGAATAGAAACTCAGGAAGGTCATGGCTAGTG |
|  |  | P238A-F | TATACAGTTTGTCACTCGCGACCTTCAAAGAGCATATAG |
|  |  | P238A-R | CTATATGCTCTTTGAAGGTCGCGAGTGACAAACTGTATA |
|  |  | P238H-F | TATACAGTTTGTCACTCCACACCTTCAAAGAGCATATAG |
|  |  | P238H-R | CTATATGCTCTTTGAAGGTGTGGAGTGACAAACTGTATA |
|  |  | T239A-F | CAGTTTGTCACTCCCGGCCTTCAAAGAGCATATAG |
|  |  | T239A-R | CTATATGCTCTTTGAAGGCCGGGAGTGACAAACTG |
|  |  | T239R-F | CAGTTTGTCACTCCCGCGCTTCAAAGAGCATATAG |
|  |  | T239R-R | CTATATGCTCTTTGAAGCGCGGGAGTGACAAACTG |
|  |  | F240A-F | GTTTGTCACTCCCGACCGCCAAAGAGCATATAGAAAC |
|  |  | F240A-R | GTTTCTATATGCTCTTTGGCGGTCGGGAGTGACAAAC |
|  |  | F240M-F | TTGTCACTCCCGACCATGAAAGAGCATATAGAA |
|  |  | F240M-R | TTCTATATGCTCTTTCATGGTCGGGAGTGACAA |
| **PTF/HRM Mutagenesis For CsUGT75L12** | 5′-terminal of PTF/HRM | CsUGT75L12-pMAL-F | TCTAGAATGCAAGCGGAGAAAGCTAGGC |
|  |  | PTF/HRM-R | GTCCCATCCTGTGGAGCATCCGACTGTATATATCTGAGCTTGAAG |
|  | 3′-terminal of PTF/HRM | PTF/HRM-F | CGGATGCTCCACAGGATGGGACAGCATATAGAAACACTTGATGCAG |
|  |  | CsUGT75L12-pMAL-R | CTGCAGCTATAAGCAATCTCCTCCAACC |
|  | For the PTF/HRM | CsUGT75L12-pMAL-F | TCTAGAATGCAAGCGGAGAAAGCTAGGC |
|  |  | CsUGT75L12-pMAL-R | CTGCAGCTATAAGCAATCTCCTCCAACC |
| **PTF/HRM Mutagenesis For TMIII** | 5′-terminal of PTF/HRM | TMIII-pMAL-F | TCTAGAATGGTGCAGCACGGCCACATCCTC |
|  |  | PTF/HRM-R | GTCCCATCCTGTGGAGCATCCGACTGTATATATCTGAGCTTGAAG |
|  | 3′-terminal of PTF/HRM | PTF/HRM-F | CGGATGCTCCACAGGATGGGACAGCATATAGAAACACTTGATGCAG |
|  |  | TMIII-pMAL-R | CTGCAGCTATAAGCAATCTCCTCCAACC |
|  | For the PTF/HRM | TMIII-pMAL-F | TCTAGAATGGTGCAGCACGGCCACATCCTC |
|  |  | TMIII-pMAL-R | CTGCAGCTATAAGCAATCTCCTCCAACC |


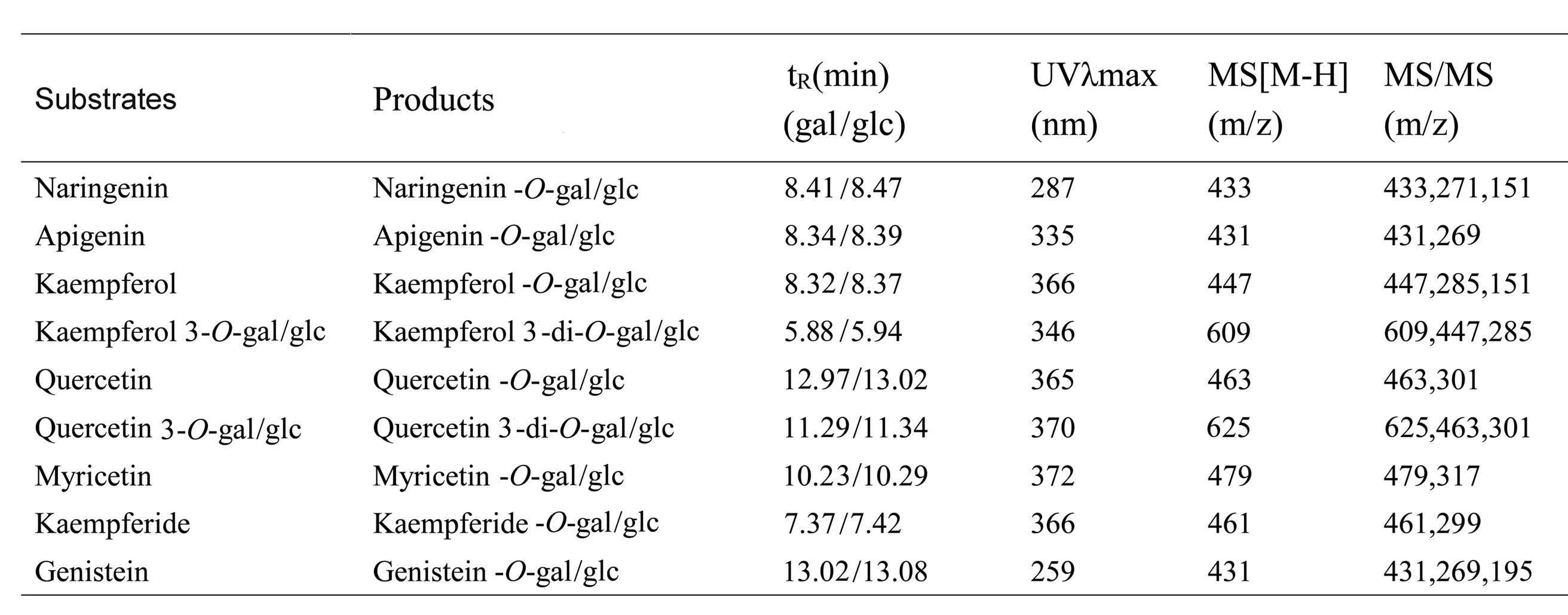


**Supplementary Table 2.** UPLC-MS/MS analysis of products from CsUGT75L12 catalysis with various substrates.

**Supplementary Table 3.** The ^1^H-NMR spectral analysis of kampferol and its monoglucosides. Kampferol and kaempferol 7-*O*-glucoside were the substrate and corresponding products of CsUGT75L12.


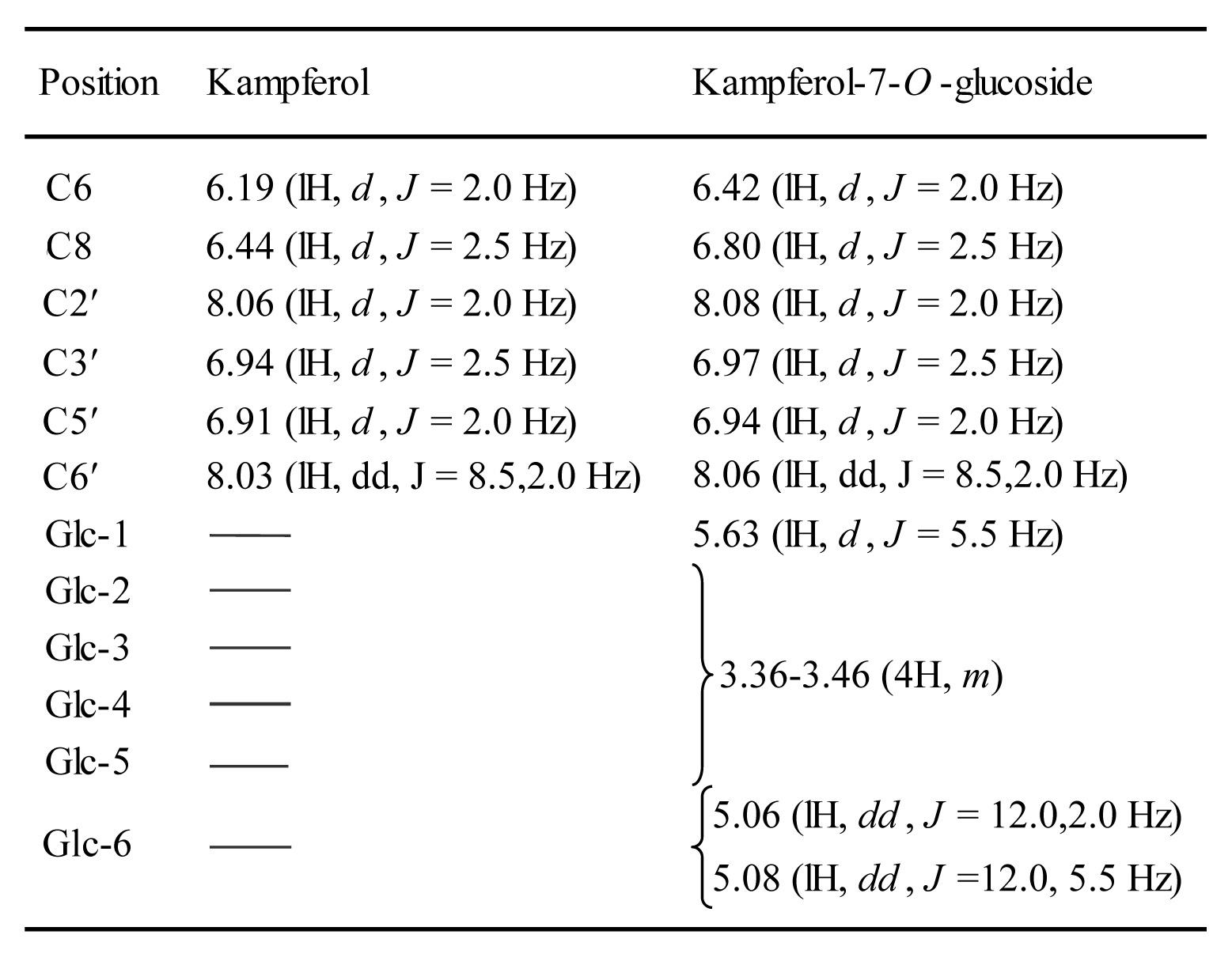


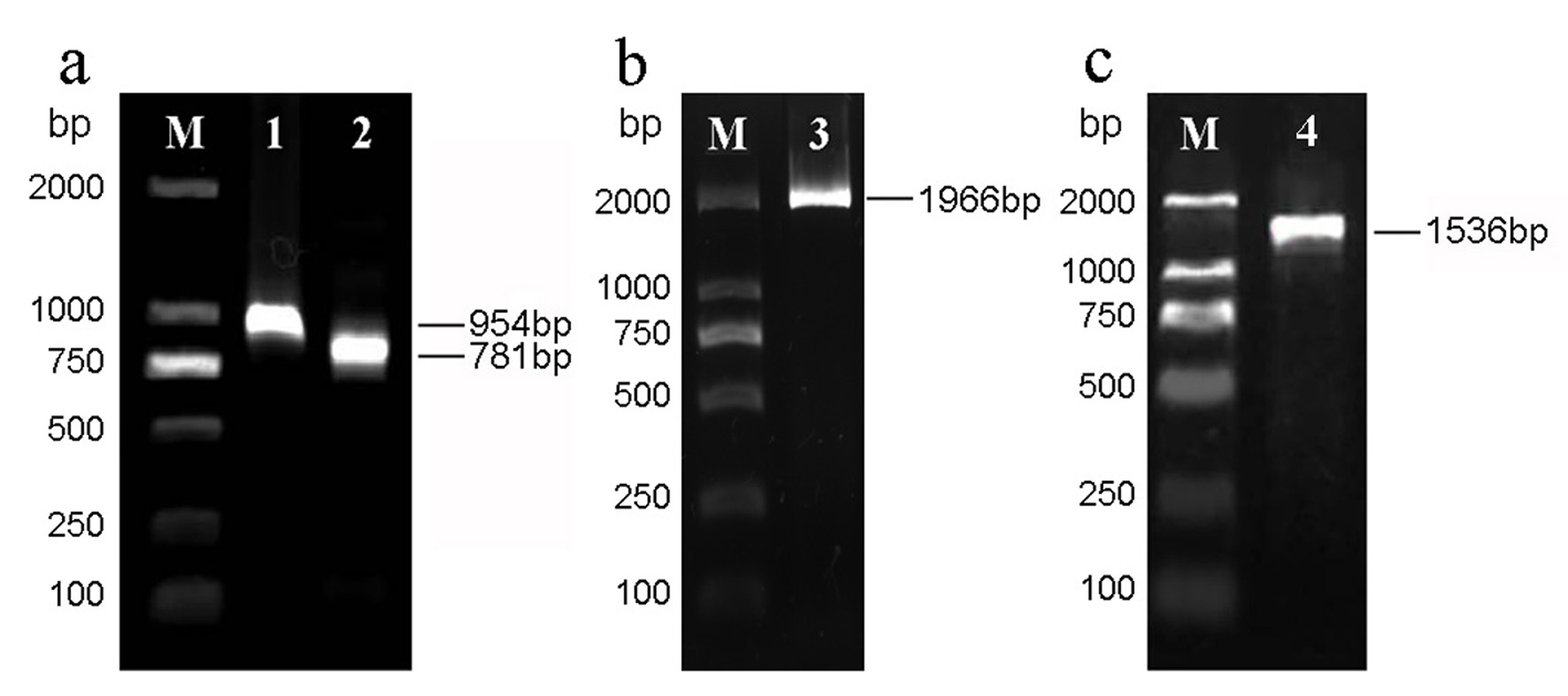


**Supplementary Figure 1.** Agarose gel analysis of the *CsUGT75L12* gene (a-c). The 1.5% agarose concentration was used in all electrophoresis analysis. M: Marker; 1: 3'Race of *CsUGT75L12*; 2: 5'Race of *CsUGT75L12*; 3: Segmental cDNA of *CsUGT75L12*; 4: Full ORF of *CsUGT75L12*.


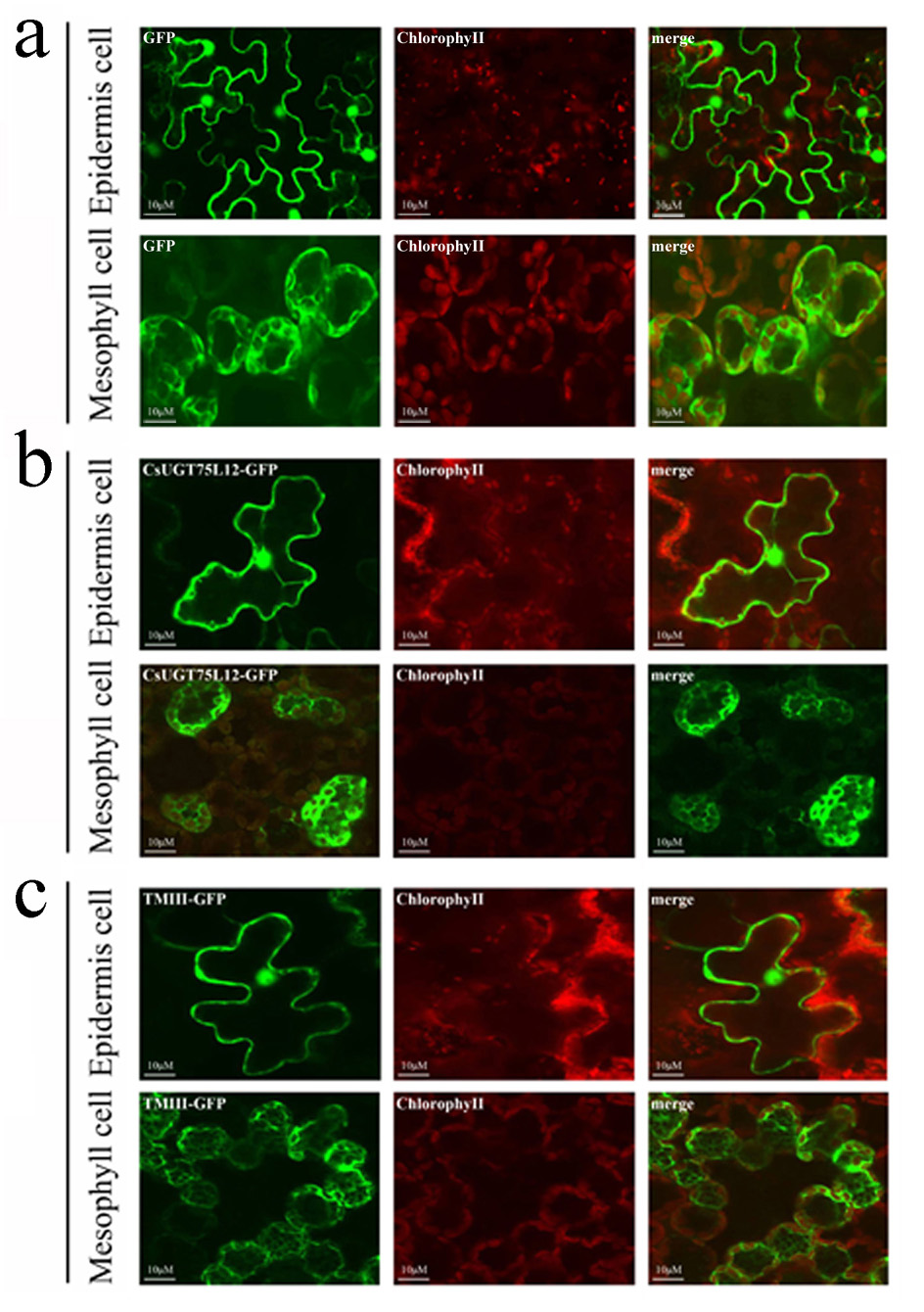


**Supplementary Figure 2.** Subcellular localization of CsUGT75L12 and TMIII in Nicotiana benthamiana leveas. Green fluorescent protein (GFP) was fused to the C-terminus of CsUGT75L12 and TMIII, respectively. (a) Expression of the control pGWB5-GFP-GFP fusion alone or merged with chlorophyll autofluorescence; (b) Expression of the pGWB5-CsUGT75L12-GFP fusion alone or merged with chlorophyll autofluorescence; (c) Expression of the pGWB5-TMIII-GFP fusion alone or merged with chlorophyll autofluorescence.


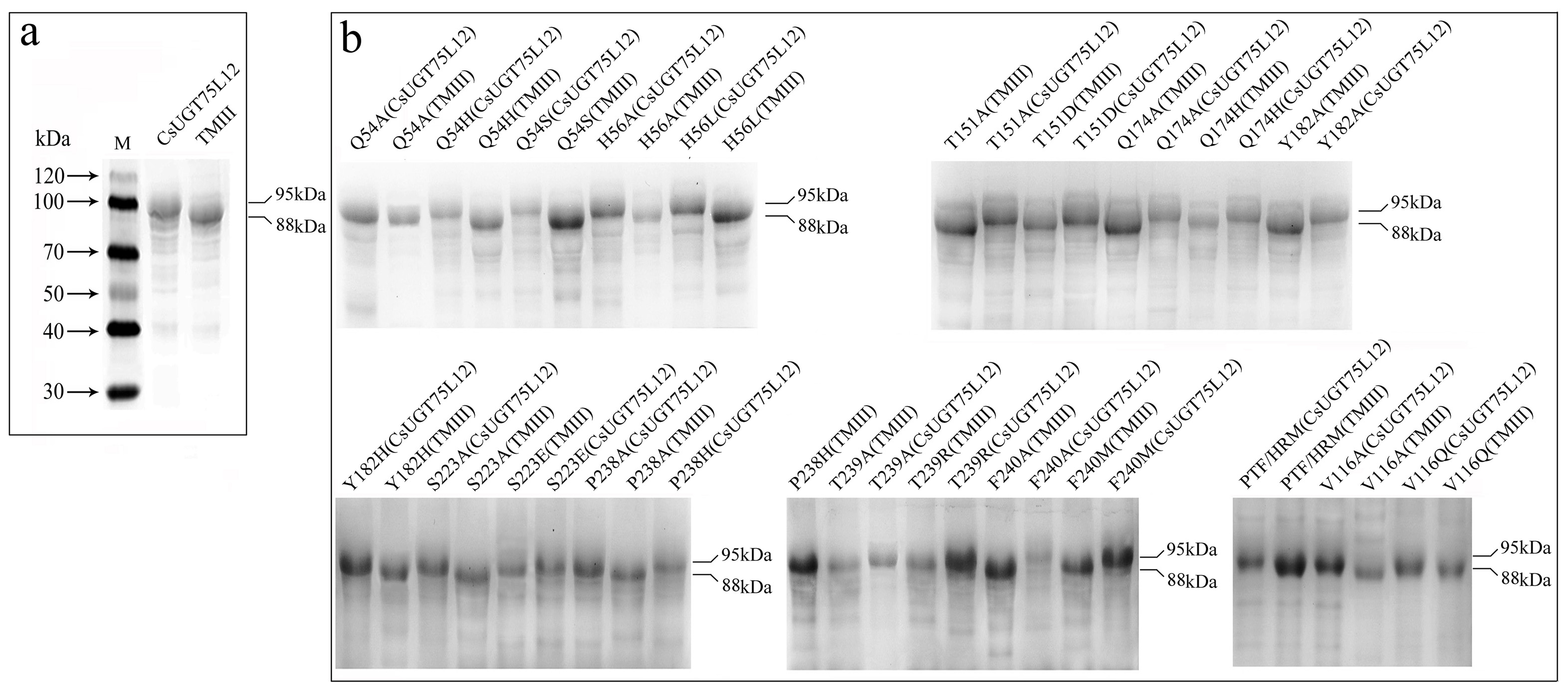


**Supplementary Figure 3.** Full length SDS-PAGE electrophoretic analysis of CsUGT75L12(a) and mutant proteins (b). The 12% polyacrylamide concentration was used in all electrophoresis analysis. These gels were imaged in a ChemiDoc^TM^ MP Imaging System (Bio-Rad, America) under the condition of frequency 50/60 Hz and max rated power 200 VA. M: Protein marker; CsUGT75L12: purified recombinant CsUGT75L12 protein (approximately 95 kDa); TMIII: purified proteinrecombinant TMIII protein (approximately 88 kDa); Various mutant proteins as follow: Q54A(CsUGT75L12), Q54A(TMIII), Q54H(CsUGT75L12), Q54H(TMIII), Q54S(CsUGT75L12), Q54S(TMIII), H56A(CsUGT75L12), H56A(TMIII), H56L(CsUGT75L12), H56L(TMIII), T151A(CsUGT75L12), T151A(TMIII), T151D(CsUGT75L12), T151D(TMIII), Q174A(CsUGT75L12), Q174A(TMIII), Q174H(CsUGT75L12), Q174H(TMIII), Y182A(CsUGT75L12), Y182A(TMIII), Y182H(CsUGT75L12), Y182H(TMIII), S223A(CsUGT75L12), S223A(TMIII), S223E(CsUGT75L12), S223E(TMIII), P238A(CsUGT75L12), P238A(TMIII), P238H(CsUGT75L12), P238H(TMIII), T239A(CsUGT75L12), T239A(TMIII), T239R(CsUGT75L12), T239R(TMIII), F240A(CsUGT75L12), F240A(TMIII), F240M(CsUGT75L12), F240M(TMIII), PTF/HRM(CsUGT75L12), PTF/HRM(TMIII), T116A(CsUGT75L12), T116A(TMIII), T116Q(CsUGT75L12), T116Q(TMIII).


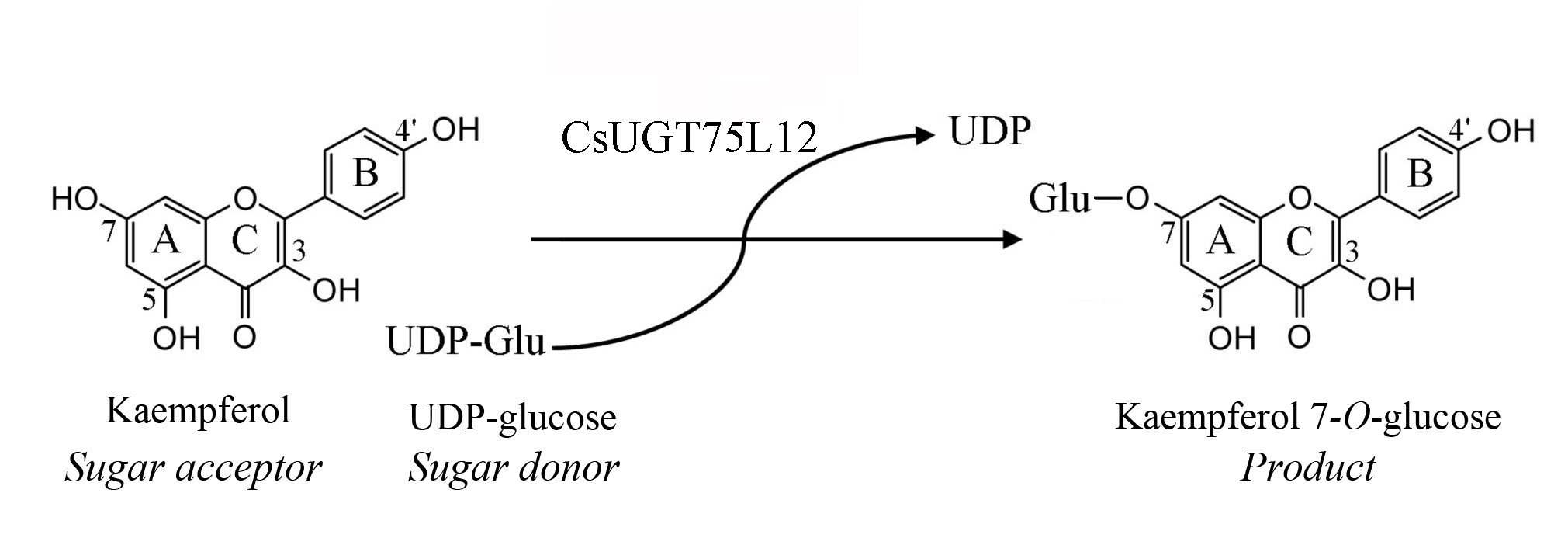


**Supplementary Figure 4.** Chemical reaction and structural formula. The CsUGT75L12 protein catalyzed the conjugation of glucose from the sugar donor, UDPG, to the 7-position of an accepter, such as kaempferol. The UDP and kaempferol 7-*O*-glucoside were enzymatic products.


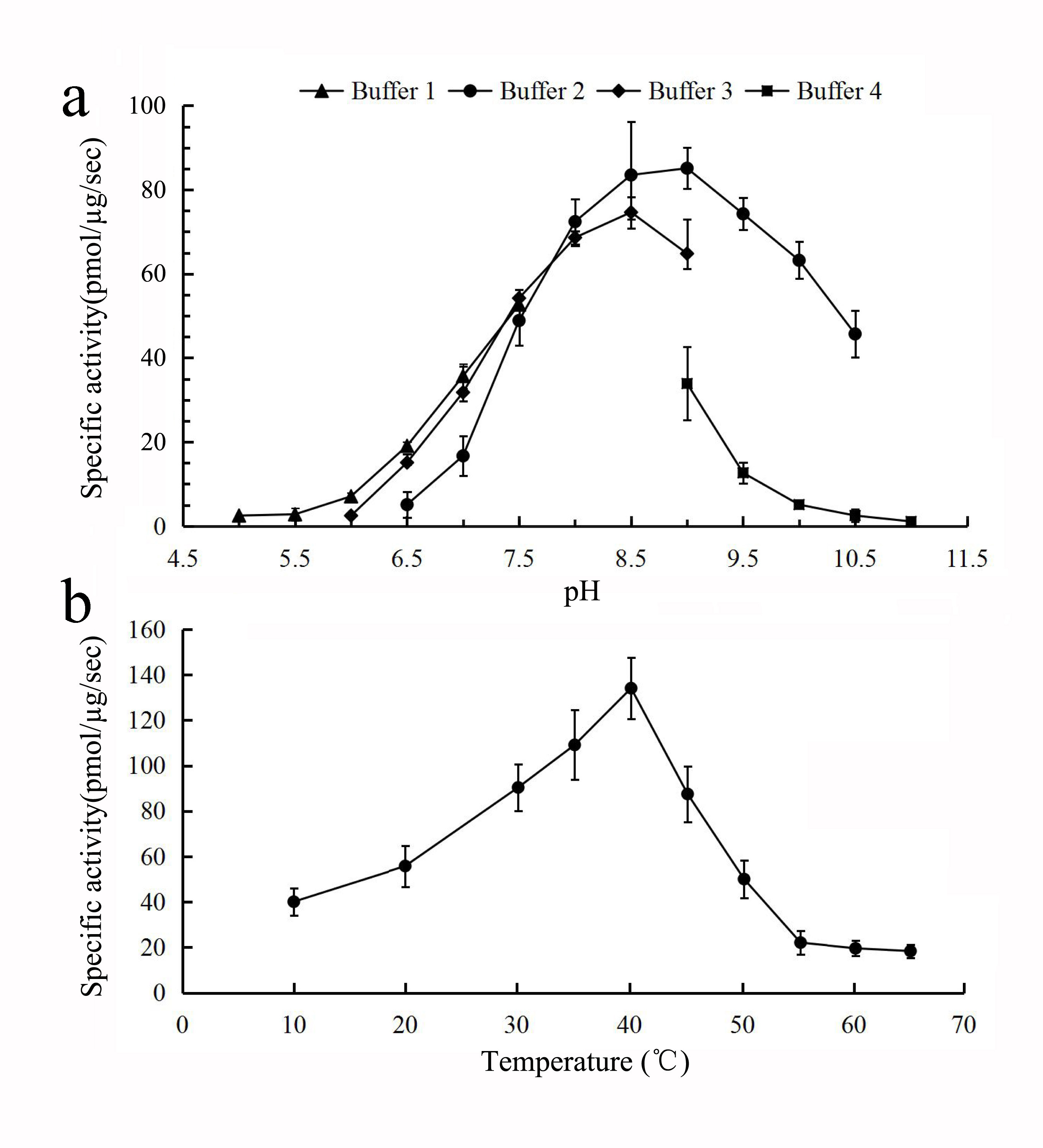


**Supplementary Figure 5.** The optimal condition of the rCsUGT75L12 reaction. Each reaction mixture (50 μl) was incubated with UDP-Glc (2.5 mM) and Kaempferol (250 μM) at different ranges of pH (4.5~11.0) and temperature (10~65℃) for 30 min. Subsequently, the reactions were terminated by adding 50 μl methanol. (a) Effect of reaction pH on rCsUGT75L12 activities, with 0.5 pH increments, at 30℃ for 30 min. (b) Effect of reaction temperature on rCsUGT75L12 activities, with 5℃ intervals, at pH 9.0 for 30 min. Data were presented as the average mean of three independent trials ± SD. Buffer 1 : 100 mM acid-sodium citrate (pH 4.0-7.5); Buffer 2 : 100 mM Tris-HCl (pH 6.5 to 8.5); Buffer 3 : 100 mM phosphate (pH 6.0 to 9.0); Buffer 4 : 100 mM Na2CO3/NaHCO3 (pH 9.0 to 11.0)


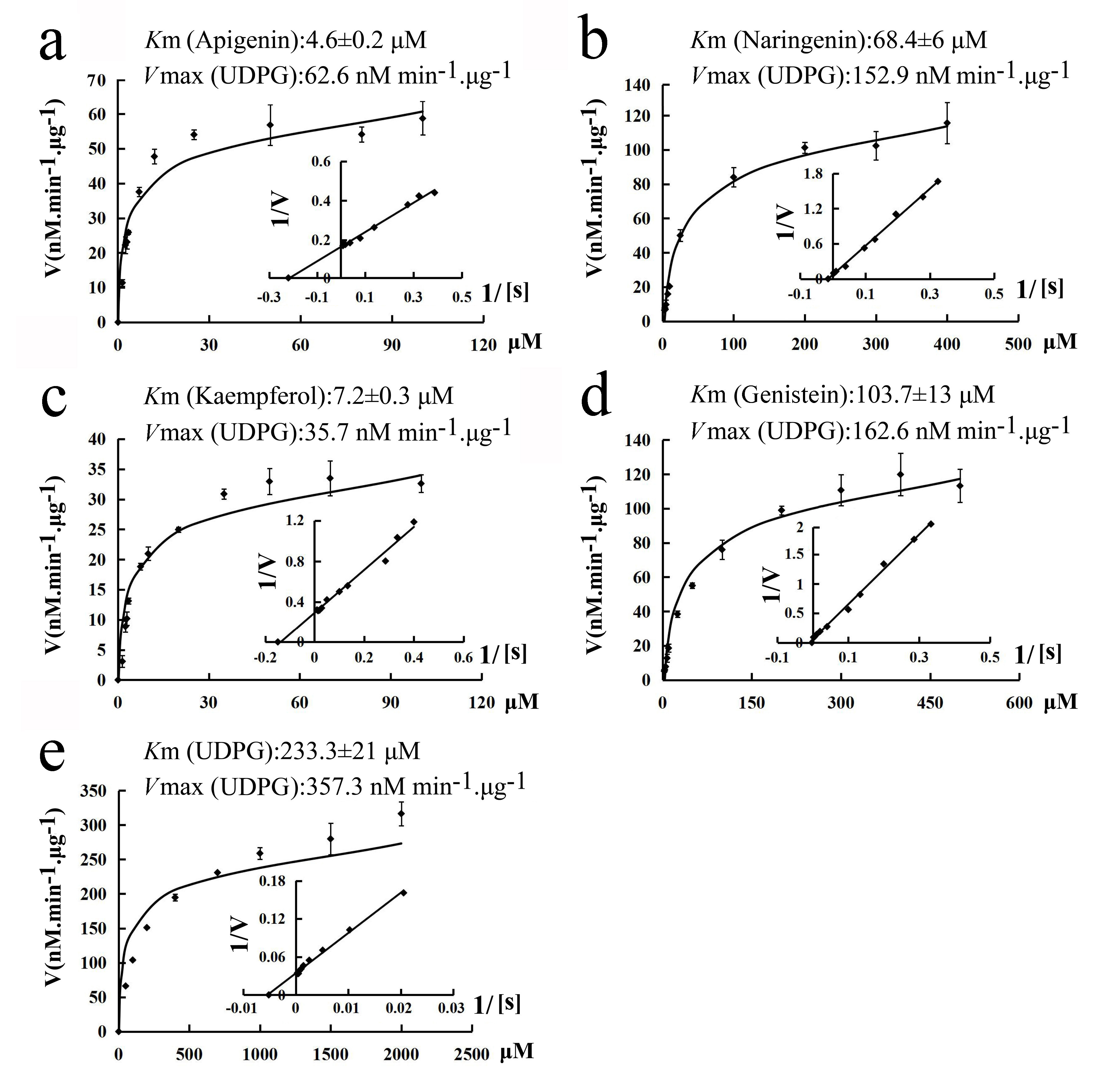


**Supplementary Figure 6.** Substrate specific analysis of rCsUGT75L12. The catalytic rate to different substrates were detected by capillary electrophoresis (CE) system and the *K*_m_ values against different substrates were calculated using hyperbolic Michaelis–Menten saturation curves. (a) Apigenin (0~100 μM) and UDP-Glc (2.5 mM); (b) Naringenin (0~400 μM) and UDP-Glc (2.5 mM); (c) Kaempferol (0~100 μM) and UDP-Glc (2.5 mM); (d) Genistein (0~500 μM) and UDP-Glc (2.5 mM); (e) UDP-Glc (0~2 mM) and Kaempferol (250 μM). Data were presented as the average mean of three independent trials ± SD.


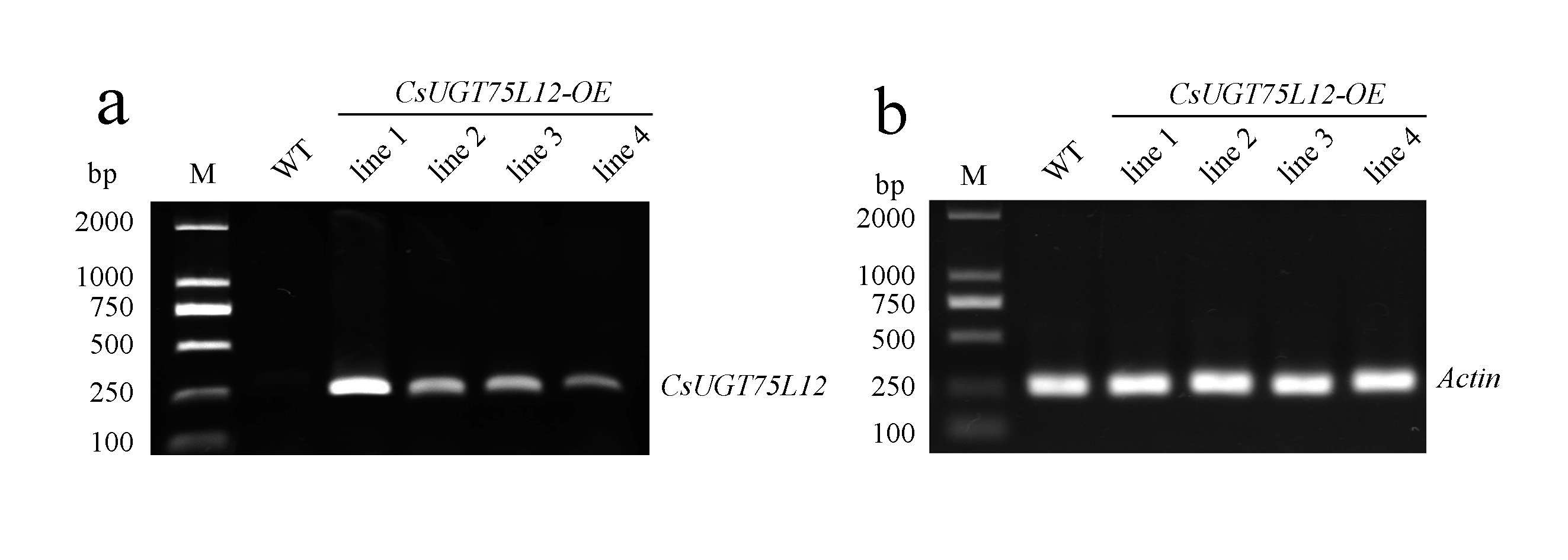


**Supplementary Figure 7.** Full length agarose gel analysis of cropped images shown in **Figure 4b**. Semi-quantitative RT-PCR of *CsUGT75L12* and *Actin* were performed under 29 cycles. (a) The expression analysis of *CsUGT75L12* in the overexpressing *CsUGT75L12* lines and wild type *Arabidopsis thaliana* (WT). (b) The expression analysis of *Arabidopsis thaliana* reference gene *Actin* in the overexpressing *CsUGT75L12* lines and WT. The 1.5% agarose concentration was used in all electrophoresis analysis.


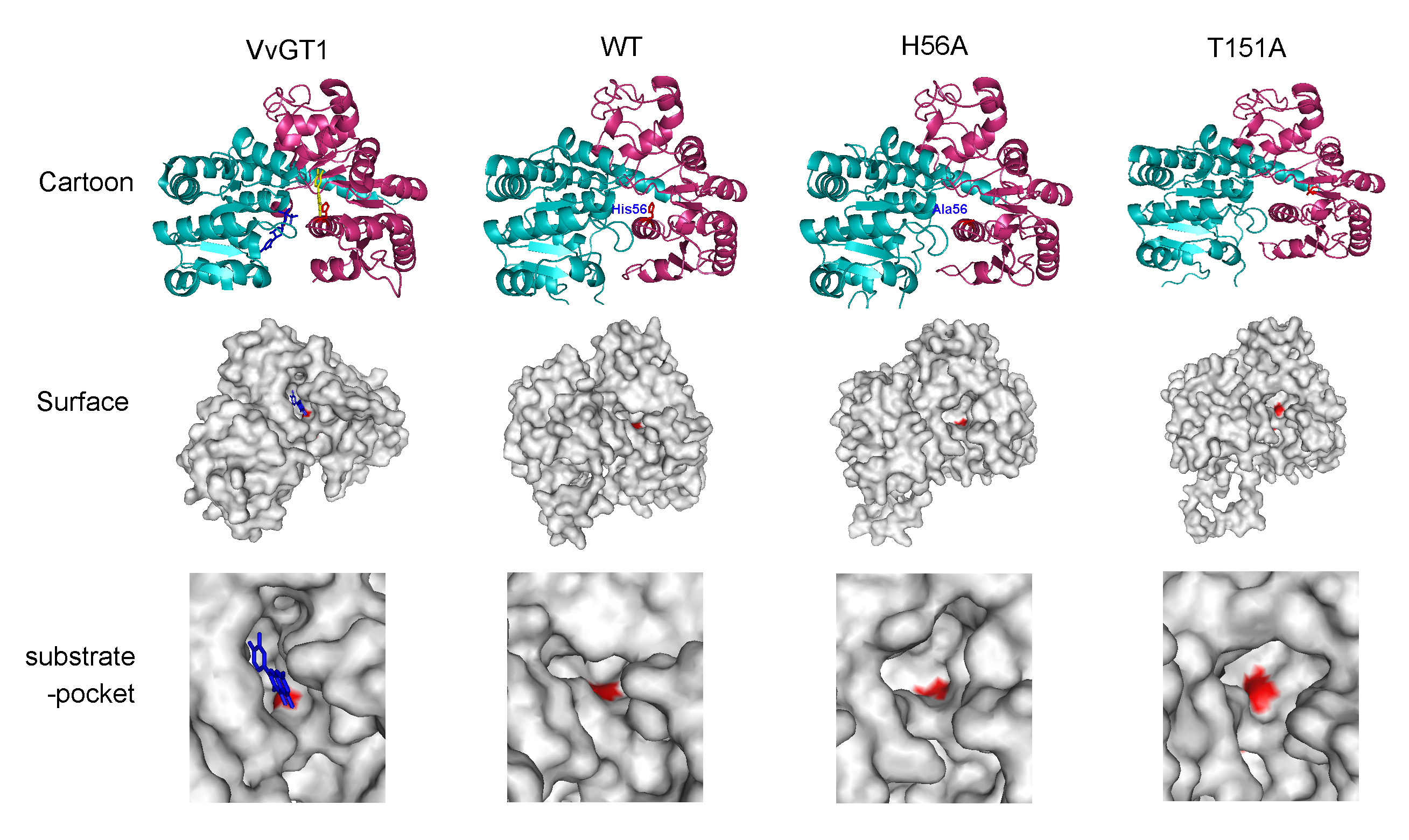


**Supplementary Figure 8.** The three-dimensional model for the both H56A and T151A mutation protein. Compared to the wild type (nature CsUGT75L12), the single substitution H56A and T151A render the substrate binding pocket of CsUGT75L12 more ‘open’. These results may explain why the catalytic activity of both the CsUGT75L12 and MTIII [increase](../../../../C:%5CDocuments%20and%20Settings%5CAdministrator%5CLocal%20Settings%5CApplication%20Data%5CYodao%5CDeskDict%5Cframe%5C20150706100352%5Cjavascript:void(0)%3B)d when two single substitution at either H56A or T151A was made, respectively.

***Accession Numbers***

Sequence data from this article can be found in the Arabidopsis Genome Initiative or Genbank/EMBL/DDBJ databases under the following accession numbers: UGT78G1（XP_003610163 ）VvGT6（NM_001280903 ）AtF3RT（NM_121711）AtF3Rht（AF360160）VvGT1（AF000371）PhF3GT（AF165148）PfF3GT（AB002818）HvF3GT(AK358129) IhA5GT(AB113664) JIGT(AB000623) SsGT1(AY033489) NtGT2(AB072919) EPGT2(AB078963) PfA5GT(pY3R4 AB013596 ) ThA5GT(AB076698) Gt5GT7(AB363839) PhA5GT( AB555731) VhA5GT (AB013598 ) UGT75L6(AB555731) GeIF7GT( AB098614) PfUGT2(AB362994) AmUGT36 (AB371297) AmUGT73N1( AB371298) PfUGT31 (AB362995) SbF7GT(AB031274) UGT73A9 (AB362993) NtF7GT(XM_009763503) UGT73A10 (AB360612) AtF73GT(NM_119576) AtF7GT(AF527807) Pf UGT57 (AB362992) GmF7GT(NM_001248232) SiUGT23 (AB362990) PfUGT50 (AB362991) AmUGTcg10 (AB362988) SlUGT1 (AB362989) ThF7GT (AB477350) UGT71G1(AAW56092) GmF3G6"RhaT(NM_001288595) CsF7G6"RhaT(ABA18631) AtA3G2"XylT (NM_124785) InA3G2"GlcT (AB192316) IpA3G2"GlcT (AB192315) CmF7G2"RhaT (AAL06646) SiF1,6GlcT AB333799
